# Strategies to improve on selection based on estimated breeding values

**DOI:** 10.1186/s12711-026-01034-z

**Published:** 2026-02-14

**Authors:** Torsten Pook, Azadeh Hassanpour, Tobias A. M. Niehoff, Mario P. L. Calus

**Affiliations:** 1 Animal Breeding and Genomics, Wageningen University and Research, P.O. Box 338, 6700 AH Wageningen, The Netherlands; 2 Department of Animal Sciences, Animal Breeding and Genetics Group, University of Goettingen, Albrecht-Thaer-Weg 3, 37075 Goettingen, Germany

## Abstract

**Background:**

Selection of individuals based on their estimated breeding values (EBV) aims to maximize response to selection in the next generation under an additive model. However, when the aim does not only include short-term population-wide genetic gain but also genetic gain over multiple generations, an optimal strategy is not as clear-cut, as maintenance of genetic diversity may become an important factor. This study provides an extended comparison of existing selection strategies in a unifying testing pipeline using the simulation software MoBPS.

**Results:**

Applying a weighting factor on estimated SNP effects based on the frequency of the beneficial allele resulted in an increase of the long-term genetic gain of 1.6% after 50 generations, while reducing inbreeding rates by 16.2% compared to truncation selection based on EBV. However, this also resulted in short-term losses in genetic gain of 1.2% with the break-even point reached after 25 generations. In contrast, inclusion of the average kinship of an individual with individuals that would be selected based on their EBVs as an additional trait in the selection index with a weight of 17.5% resulted in no short-term losses and increased long-term genetic gain by 4.3%, while reducing inbreeding by 15.8%. Combining multiple diversity management strategies, with weights for each strategy optimized using an evolutionary algorithm, resulted in a breeding scheme with 5.1% greater genetic gain and 37.3% lower inbreeding rates than selection based on EBVs. The proposed combined strategy included the use of optimum contribution selection, weighting of SNP effects based on allele frequency, average kinship as a trait in the selection index, avoiding matings between related individuals, and lowering the proportion of selected individuals.

**Conclusions:**

The combination of strategies for the management of genetic diversity in a breeding program was shown to be far superior to the use of any singular method tested in this study. As the use of strategies for management of genetic diversity and inbreeding does not necessarily lead to short-term losses in genetic gain and comes at no extra costs, it is critical for breeding companies to implement such strategies for long-term success.

**Supplementary Information:**

The online version contains supplementary material available at 10.1186/s12711-026-01034-z.

## Background

Although there are substantial differences between livestock and plant breeding species, the general goal of breeding to improve desired traits or characteristics in the organism under selection is universal. To this end, selection of individuals to be used as parents for the next generation is of critical importance, with selection on estimated breeding values (EBVs) maximizing response to selection from the current to the next generation.

Although the importance of genetic diversity in a breeding program is a generally accepted fact (e.g., Breeder’s Equation [1]), diversity management is usually associated with reduced genetic gains in the short-term, which for a breeding company can be associated with a lower quality product in the short-term and the associated risk of going out of business when putting too much emphasis on maintaining genetic diversity. However, increasing signs of reduced heritabilities [2, 3], increased genetic load [4, 5], and increased expression of inbreeding depression [6] are reported in practice, which increases the need for more efficient management of genetic diversity without compromising short-term genetic gain too much.

To assess and compare breeding programs, stochastic simulation provides a powerful tool by generating a digital twin of a breeding program, which can then be used to precisely analyze the impact of concrete changes in breeding program design. Compared to the use of deterministic calculations based on quantitative genetics theory [7], stochastic simulation comes with the disadvantages of higher computing costs and each simulation resulting in only one realization of a stochastic process instead of an expected value. However, these downsides are compensated by the added flexibility of stochastic simulation – particularly as there is usually no deterministic model to analyze the exact impact of subtle changes on the outcomes of a breeding program. Software programs for stochastic simulation of breeding programs include MoBPS [8], AlphaSimR [9, 10], Adam [11], and QMsim [12].

Concepts for the management of genetic diversity are multifaceted, both in complexity and method, with theoretical optimal solutions often being impractical or overly complex to implement. In the following paragraphs, we will provide a general overview of different approaches that have been proposed. The focus here is on optimizing the selection step and selection criteria, and not on restructuring the breeding program design, introducing outcross material [13, 14], or on splitting the breeding population into subgroups [15, 16].

The gold standard in livestock breeding is the use of optimum contribution selection (OCS) [17] to determine the contribution of each individual to the subsequent generation to maximize genetic gain for a target inbreeding rate or to minimize the inbreeding level for a target genetic gain. Concepts of OCS can be combined with minimum coancestry mating [18, 19] to mate individuals to genetically more distant partners to further reduce inbreeding in the progeny generation. However, in reality, implementation of these concepts in their entirety can be difficult. For example, it is usually not possible to have a single offspring from a sow, as a litter contains multiple individuals, resulting in more or fewer offspring than is theoretically optimal. Similarly, in plant breeding, logistical limitations and field design may require simplifying a theoretically optimal strategy. Simplified strategies include selecting a maximum number of individuals from a given family or avoiding matings between close relatives.

Over the years, a variety of alternatives to OCS have been proposed based on various objectives and/or theoretical motivations. For instance, Goddard [20] suggested putting more emphasis on rare SNP alleles when computing genomic EBVs, as they are more likely to get lost due to drift, hitchhiking, or unfavorable associations with closely linked other quantitative trait loci (QTLs). Building upon the work of Goddard [20], Jannink [21] suggested a simplified weighting factor of ${p}^{-\frac{1}{2}}$ on SNP effect estimates when computing genomic EBVs, where $p$ is the frequency of the beneficial variant. Liu et al. [22] demonstrated the long-term benefits of this approach in simulation.

As selection of individuals in a breeding program is typically done based on multiple traits that are combined in a selection index, another strategy to balance short-term genetic gain and inbreeding is to include the contribution of an individual to the genetic diversity of the population as a pseudo-trait in a selection index. An example of this can be found in the Net Merit index used in US dairy cattle breeding [23].

In addition to the expected genetic value of hypothetical offspring of a selection candidate, i.e., its breeding value, the variance of breeding values of hypothetical offspring can also be considered in selection decisions. Hence, an individual with a lower breeding value but higher variance of breeding values among offspring can still be of value when the goal is to maximize the probability of generating some offspring with extreme breeding values. The Mendelian sampling variance (MSV) of gametes produced by a selection candidate plays a key role in this context. Several approaches to integrate MSV into breeding strategies have been proposed, but the primary aim is to improve short-term gain genetic [24–29]. However, some studies also highlight the long-term benefits of integrating MSV, such as enhanced genetic gain and diversity, with Allier et al. [30, 31] specifically targeting long-term “usefulness” of individuals in breeding decisions.

Lastly, approaches such as those implemented in the software programs MateSel [32] and AlphaMate [33] can be used to solve the OCS problem using an evolutionary algorithm and then generate a concrete mating plan. A potential benefit from the use of such approaches is that it is much easier to implement restrictions or constraints on the selection of individuals, e.g., to select a certain number of individuals that will all have the same number of litters, compared to the traditional deterministic solution of OCS based on Lagrange optimization, which just specifies the optimal contribution of all selection candidates [17].

Most studies on strategies to maintain genetic diversity in breeding programs compare their proposed approach only against straight selection on EBVs rather than against other previously proposed strategies for maintenance of genetic diversity. Thus, the goal of this study was to compare approaches that have been proposed in the literature for management of genetic diversity with each other. In addition, an evolutionary optimization pipeline [34] was employed to assess whether and how proposed approaches can be combined and provide recommendations on methods to use in real-world breeding programs. A further aim was to provide a uniform testing pipeline for breeders and researchers to easily compare new approaches with common standards in the field, including the approaches evaluated in this study.

## Methods

A generic breeding program was simulated as a baseline breeding program for subsequent optimization using the R-package MoBPS [8]. In brief, a genome with 10 chromosomes of 2.5 Morgan length and 25,000 equidistantly spaced SNPs across the genome was simulated. A quantitative trait with 1,000 additive QTLs and a heritability of 0.3 was simulated, with the QTLs being a subset of the SNPs. To initialize the population with a realistic population structure, 1,000 founder individuals with randomly sampled genotypes were generated, followed by 10 generations of mating with phenotypic selection as a burn-in phase. Subsequently, 50 generations of breeding were simulated using truncation selection among 1000 selection candidates per generation and a 1:1 sex ratio. Each generation, the top 40 males and top 100 females were selected as sires and dams to generate the breeding population of the next generation. The selected individuals had equal contributions to the next generation. Selection was on EBV derived by genomic best linear unbiased prediction (GBLUP) [35, 36] that included only individuals from the current generation, with all individuals genotyped and phenotyped before selection. To evaluate the output of the breeding program, an additional cohort of production individuals was generated, using sires and dams selected based on their EBVs.

Various modifications to the breeding program that will be discussed in the following subsections were evaluated based on the average outcomes across 50 replicates (unless indicated otherwise). Genetic gain in each generation was evaluated based on the average underlying true genetic value of the production individuals based on their genotypes and true effects of the QTL. Genetic variance in each generation was computed based on the true genetic values, and inbreeding levels of selection candidates in the breeding population were derived using identity-by-descent [37], as the originating founder for each chromosome segment was tracked in the simulation [8].

### Weighting allele effects by allele frequency

Following the selection strategies suggested by Goddard [20] and Jannink [21], a modified selection criterion $\widehat{{g}_{i}}$ in which marker effects are weighted based on the allele frequency of the beneficial allele in the population was considered:

1 $$\widehat{{g}_{i}}={\sum }_{j}{z}_{i,j}{w}_{j}{\widehat{a}}_{j},$$

With ${\widehat{a}}_{j}$ being the estimated effect of marker $j$, ${z}_{i,j}$ being the genotype of individual $i$ at marker $j$ and ${w}_{j}$ being the weighting factor of marker $j$. The weighting factors were calculated based on the frequency of the beneficial allele ${p}_{j}$. The following weightings were evaluated:

2 $${w}_{j}=\frac{\frac{\pi }{2}-\mathrm{arcsin}\left(\sqrt{{p}_{j}}\right)}{\sqrt{\left(1-{p}_{j}\right){p}_{j} }} , \left(Goddard\right),$$

3 $${w}_{j}={p}_{j}^{-\frac{1}{2}} , \left(Jannink\right),$$

4 $${w}_{j}={p}_{j}^{-\frac{1}{3}},$$

5 $${w}_{j}={p}_{j}^{-\frac{1}{4}},$$

6 $${w}_{j}=\mathit{min}\left(5, {p}_{j}^{-\frac{1}{2}}\right),$$

7 $${w}_{j}=d+\left(1-d\right){{p}_{j}}^{-\frac{1}{2}} for\;0\le d\le 1$$

**Fig. 1:**
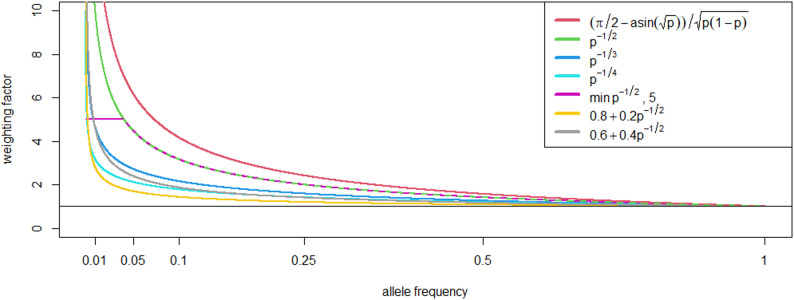
Weighting factor of estimated SNP effects based on the used allele frequency weighting functions. Weighting factor for the different functions depending on the allele frequency of the beneficial allele, with the x-axis on $\mathrm{log}(x+1)$ -scaled

The first two weighting factors follow Goddard [20] and Jannink [21], while the remaining weighting factors apply more moderate weighting of beneficial rare alleles to limit short-term losses (Fig. 1).

### Relatedness to the breeding population

Following the approach of the Net Merit index [23], relatedness of the selection candidate *i* to the breeding population, ${f}_{i}$, was incorporated as an additional trait in the selection index as:

8 $$\widehat{{g}_{i}}=-{w}_{f}{f}_{i}+\left(1-{w}_{f}\right){\sum }_{j}{z}_{i,j}{\widehat{a}}_{j}$$

Two definitions of ${f}_{i}$ were considered:

1. Average kinship to the current breeding population
2. Average kinship to top individuals, defined as individuals from the current breeding population that would be selected based on their EBVs

The weighting factor ${w}_{f}$ was chosen to yield a relative weighting between 5 to 40% for ${f}_{i}$ after standardizing both EBVs and ${f}_{i}$ to have mean zero and unit variance across selection candidates. In a multi-trait context, ${f}_{i}$ can be treated as an additional trait in the selection index. Pairwise kinships were calculated from pedigree information with a pedigree depth of seven.

### Avoiding matings between related individuals

Different levels of mating control were considered. First, matings between full- and half-sibs were avoided, as these would result in high inbreeding levels of progeny. To extend beyond these extreme cases, we propose a novel, more general strategy to avoid matings that would lead to high inbreeding levels of progeny. For each potential mating, the expected inbreeding level of a hypothetical offspring was calculated based on the pedigree-based kinship of its parents, and mating pairs exceeding a specific threshold were excluded. As the threshold, quantiles of the resulting expected inbreeding levels across all potential sire-dam pairs were considered, ranging from the 10% to the 90% quantile. For example, in a population with 40 sires and 100 dams, the 90% quantile would exclude 4,000 of the 40,000 potential sire-dam combinations. Final matings were randomly sampled from the set of allowed mating pairs. Note that this could lead to minor uneven contribution among selected individuals, e.g., if an individual’s hypothetical progeny had a high expected inbreeding level when mated to a particularly high share of individuals of the other sex. Overall, differences in contributions among individuals with this approach are, however, expected to be small.

As an alternative criterion, the expected kinship of a potential offspring to the current generation of the breeding population was used to identify matings to avoid. Note that the expected kinship of a hypothetical offspring directly corresponds to the mean kinships of its parents to the current generation. Therefore, this approach strongly reduced contributions of sires and dams with high average kinship to the current generation and also resulted in uneven contributions among selected individuals.

### Optimum contribution selection

For the use of optimum contribution selection [17], the R-package optiSel [38] was used to determine optimal individual contributions by solving the traditional deterministic optimization problem under a fixed threshold on the increase in average kinship from the current to the next generation. To mimic the common restriction of animal breeding programs that contributions on the female side cannot easily be controlled, OCS was applied only to derive contributions for male selection candidates [38]. For this purpose, optiSel was provided with information on all male candidates and the already selected females, with the objective of maximizing genetic progress (using target = “max.BV”, uniform = “female” in optiSel), while restricting the increase in average kinship to the reference population. Threshold values for the allowed increase in average kinship ranged from 0.3 to 0.7%. A pedigree-based relationship matrix with a pedigree depth of 7 was used to derive kinships.

As an alternative approach, the software AlphaMate [33] was used to solve the optimum contribution selection problem via evolutionary optimization, in which average kinship among selected individuals was penalized rather than constrained by a hard threshold. AlphaMate suggested 1000 matings from 40 males and 100 females, with equalized contributions across selected individuals (using EqualizeMaleContributions = Yes, EqualizeFemaleContributions = Yes in AlphaMate) to maintain the same selection proportions as the baseline scenario. To balance genetic gain and genetic diversity, the angle parameter $\theta$ in AlphaMate was varied from 5 to 45°, where 0° corresponds to selection solely for genetic gain and 90° to selection solely for genetic diversity. For intermediate angles, the effective weighting aims to achieve approximately $\mathrm{cos}\left(\theta \right)$ of the maximal attainable genetic gain and $\mathrm{sin}(\theta )$ of the minimum attainable kinship among selected individuals, although evolutionary optimization under additional constraints can deviate from this idealized circular frontier. To reduce computing time, the convergence criterion and the maximum number of iterations were set to nEvolAlgNumberOfIterations = 1000 and nEvolAlgNumberOfIterationsStop = 50, respectively. A genomic relationship matrix derived using van Raden (method 1) [36] was used. Allele frequencies estimated from the selection candidates were used to center genotypes in the computation of the genomic relationship matrix.

The two considered OCS approaches differed in several aspects: choice of relationship matrix (pedigree vs. genomic), the nature of the inbreeding constraint (fixed threshold vs. penalized), mate allocation (not performed vs. performed), selection of females (based on EBVs vs. as part of the optimization), and contribution (different sires with different contributions vs. all sires with the same contributions). These differences do not inherently reflect deterministic versus evolutionary solutions to the OCS problem; rather, they represent two complementary strategies of OCS. In principle, all of these aspects can be accommodated within both the deterministic and stochastic framework.

### Mendelian sampling variance

To assess the potential benefits of accounting for MSV in selection decisions, several previously proposed criteria were evaluated. Bijma et al. [25] theoretically motivated indices to select based on the probability of hypothetical offspring of an individual to be selected (${I}_{5}$, [25]) and to maximize response to recurrent selection (${I}_{6}$, [25]) were both considered:

9 $${\widehat{g}}_{i}^{{I}_{5}}= \widehat{{A}_{i}}+\sqrt{2} {x}_{p}{\sigma }_{{\widehat{g}}_{i}},$$

10 $${\widehat{g}}_{i}^{{I}_{6}}= \widehat{{A}_{i}}+\sqrt{2} {i}_{p}{\sigma }_{{\widehat{g}}_{i}},$$

where ${\widehat{A}}_{i}$ denotes the genomic EBV of individual i, ${\sigma }_{{\widehat{g}}_{i}}$ is the standard deviation of the genomic EBVs of an individual’s gametes, ${i}_{p}$ is the selection intensity corresponding to a selection proportion $p$, and ${x}_{p}$ is the truncation point of the Gaussian distribution for the *p*-percent quantile.

In addition, the ExpBVSelGrOff criterion proposed by Niehoff et al. [29] was evaluated. This criterion was designed to maximize genetic response in the grand offspring and can be interpreted as an extension of ${\widehat{g}}_{i}^{{I}_{6}}$ by explicitly considering two generations instead of one. For each individual, the selection criterion was calculated as the average expected breeding value of hypothetically generated and selected grand-offspring across all possible mating combinations. Further details on the estimation procedure of ExpBVSelGrOff and MSV are in Niehoff et al. [29].

Because prediction accuracies for MSV are generally low, an additional scenario was evaluated in which individuals from the previous six generations were included in the estimation of SNP effects, rather than just the current generation, in order to improve the accuracy of MSV predictions.

### Adapted selection proportions

As a final strategy, the impact of adjusting the selection proportions was evaluated. Specifically, between 28 and 50 sires and 70 and 125 dams were selected per generation, while maintaining a selection ratio of 2:5 (40 / 100) to maintain the relative selection pressure between sexes. Although characterizing the impact of this strategy by differences in selection intensity would be appropriate if this modification was applied in isolation, the term selection intensity was avoided throughout this manuscript because, when combined with other selection strategies, selecting fewer individuals does not necessarily imply a higher selection intensity when individuals with lower EBVs are selected.

### Joint optimization

In addition to considering the proposed selection strategies individually, the combined application of multiple strategies was also investigated, as complementary approaches may yield additional benefits. To optimize the breeding program under the joint use of multiple strategies, an evolutionary algorithm based on the pipeline suggested by Hassanpour et al. [34] was applied.

In short, a set of potential breeding program designs was generated, with each design defined by a set of design parameters (Table 1). The performance of each breeding program design was evaluated using stochastic simulation, after which the most promising designs were identified and used to generate a new set of potential breeding program designs for subsequent evaluation. This procedure was iteratively repeated until convergence was reached or, at a minimum, until further changes in the target function became negligible.

**Table 1:** Overview of parameters considered in the joint optimization pipeline

Parameter	Variable name	Initialization range
Weighting factor for the beneficial alleles $= \frac{\frac{\pi }{2}-\mathrm{arcsin}\left(\sqrt{p}\right)}{\sqrt{\left(1-p\right)\cdot p}}$	${x}_{1}$	0–0.2
Weighting factor for the beneficial alleles $={p}^{-\frac{1}{3}}$	${x}_{2}$	0–0.2
Weight on average relatedness to top individuals	${x}_{3}$	0–0.2
Weight on average relatedness to breeding population	${x}_{4}$	0 –0.2
Number of selected females	$40*{x}_{5}$	0.7–1.3 (28–52)
Number of selected males	${100*x}_{5}$	0.7–1.3 (70–130)
Quantile to avoid matings based on expected inbreeding	${x}_{6}$	0.4–1.0
Quantile to avoid matings based on expected kinship	${x}_{7}$	0.9–1
Increase of average kinship in optimal contribution selection	${x}_{8}$	0.35 to 0.65%
Binary on whether deterministic optimal contribution selection is used?	${x}_{9}$	FALSE / TRUE

To evaluate the performance of a given breeding program design, a target function that incorporates short- and long-term genetic gain and maintenance of genetic diversity was defined. Genetic gain was quantified using the average true genetic value ${\overline{g} }_{t}$ of the breeding population in generation $t$, with equal weights assigned to each generation. Because ${\overline{g} }_{t}$ represents the cumulative genetic gain up to generation $t$, genetic gain achieved in earlier generations contributed more strongly to the objective function than genetic gain in later generations. When interpreting ${\overline{g} }_{t}$ as a production trait, the sum of the ${\overline{g} }_{t}$ across generations could be viewed as the total production from generation 1 to 50. Genetic diversity was quantified using the average inbreeding level in generation 50, ${\overline{f} }_{50}$, and the genetic variance after 50 generations, ${\sigma }_{{\overline{g} }_{50}}^{2}$. As these three components were measured on different scales, each term was first scaled by 25, 0.045, and 0.3, respectively, which approximately reflect the standard deviations of each factor across evaluated scenarios. Genetic gain was assigned a relative weight of 2/3, while each of the two genetic diversity metrics was given a weight of 1/6, resulting in the following target function:

11 $$m=\frac{2}{3}\cdot \frac{1}{25}{\sum }_{t=1}^{50}{\overline{g} }_{t}+\frac{1}{6}\cdot \frac{9}{200}{\sigma }_{{\overline{g} }_{50}}-\frac{1}{6}\cdot \frac{3}{10}\cdot {\overline{f} }_{50}.$$

The chosen weighting and scaling factors for this target function are more or less arbitrary and do not aim to mimic a concrete breeding program or objective, as determining appropriate scaling factors and defining a suitable breeding objective can be key challenges in practice and will be highly application-specific [34, 39].

In this approach, a total of nine parameters were subject to optimization, as summarized in Table 1. This included four parameters ($${x}_{1},{x}_{2},{x}_{3},{x}_{4})$$ to control the weighting of estimated SNP effects based on Goddard [20], a more moderate weighting on $${p}^{-\frac{1}{3}}$$, the relative weighting on the average relatedness of the candidate to top individuals $$({f}_{i,1})$$ and to the full population $$({f}_{i,2})$$, resulting in the following formula for the selection criterion:

12 $$\begin{aligned} \widehat{{g_{i} }} = & x_{1} \sum _{j} z_{{i,j}} \frac{{\frac{\pi }{2} - \arcsin \left( {\sqrt p } \right)}}{{\sqrt {\left( {1 - p} \right) \cdot p} }}\widehat{{a_{j} }} \\ & \quad + x_{2} \sum _{j} z_{{i,j}} p^{{ - \frac{1}{3}}} \widehat{{a_{j} }} - x_{3} f_{{i,1}} - x_{4} f_{{i,2}} \\ & \quad + \left( {1 - x_{1} - x_{2} - x_{3} - x_{4} } \right)\sum _{j} z_{{i,j}} \widehat{{a_{j} }}. \\ \end{aligned} $$

As an example, ${x}_{2}= {x}_{3}= {x}_{4}=0 \;\mathrm{and} \;{x}_{1}=0.1$ corresponds to a selection criterion that places 90% weight on the standard EBV of an individual and 10% on the allele-frequency-weighted selection criterion suggested by Goddard [20], with no weight on the other three selection criteria.

In addition to these four parameters, a parameter to jointly control the selection proportions of males and females ${(x}_{5})$ was considered, as well as two parameters to control what share of matings were avoided based on expected inbreeding (${x}_{6}$) and kinship (${x}_{7}$). Finally, a binary parameter to control whether OCS using optiSel was used on the male side (${x}_{8}$) and if so, an additional parameter (${x}_{9}$) to specify the allowed increase in average kinship.

For the evolutionary algorithm [34], the binary parameter was initialized by sampling from a Bernoulli random variable with a probability of 0.5, while all continuous parameters were initialized from a uniform distribution with initialization ranges given in Table 1. No budgetary or operational constraints were applied that would require scaling or a more complex initialization scheme.

As a second variant of the joint optimization pipeline, a modified target function was used to place greater emphasis on short-term genetic gain. To this end, genetic gain in each generation was discounted using an interest factor (3% per generation) to reflect the relative value of genetic gain to generation 0 [40]. Additionally, the scaling factor for genetic gain was increased by a factor of 2.5 to compensate for the reduced variation in the discounted genetic gain:

13 $$m=\frac{2}{3}\cdot \frac{1}{10}{\sum }_{t=1}^{50}\frac{{\overline{g} }_{t}}{{1.03}^{t} }+\frac{1}{6}\cdot \frac{9}{200}{\sigma }_{{\overline{g} }_{50}}-\frac{1}{6}\cdot \frac{3}{10}{\overline{f} }_{50}.$$

Because the evolutionary pipeline by Hassanpour et al. [34] did not always reach convergence, the final optimum was determined based on visual inspection from the final iterations. The resulting breeding scheme was subsequently evaluated using stochastic simulation with the optimized parameters, with 50 replicates.

## Results

**Fig. 2:**
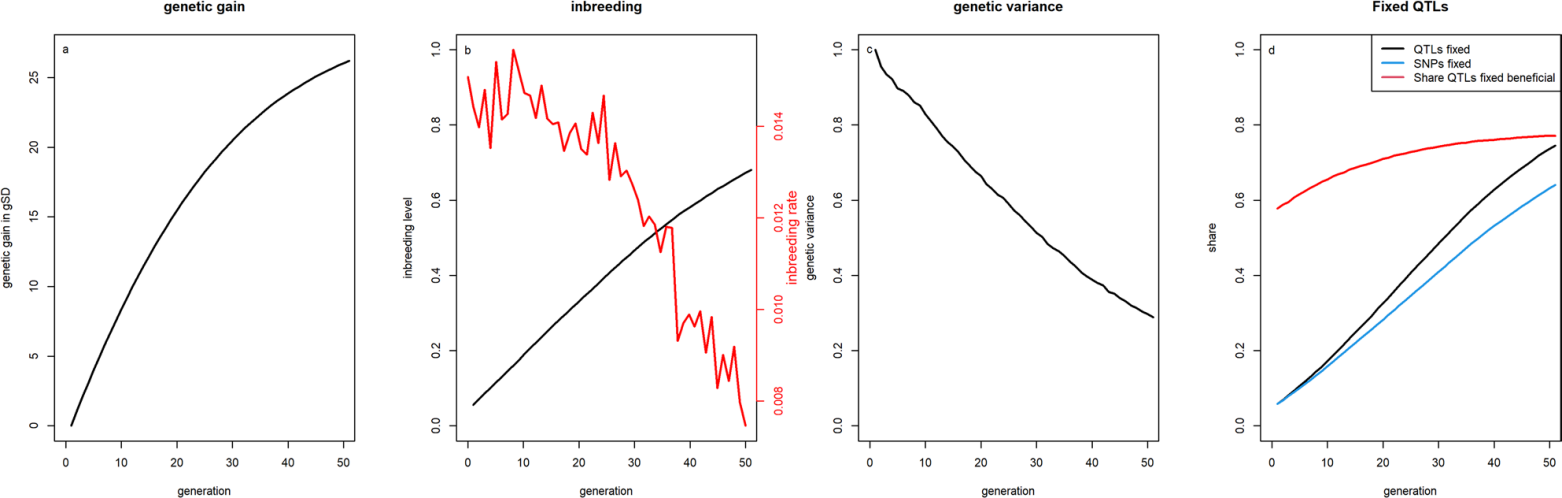
Genetic gains, inbreeding levels, genetic variance and proportion of fixed QTLs in the baseline scenario. Genetic gains (**a**), inbreeding levels and rates (**b**), genetic variance (**c**), and share of SNPs and QTLs fixed (**d**) for the baseline scenario with selection based on estimated breeding values

In the baseline scenario, a genetic gain of 26.2 genetic standard deviations (gSD) was obtained after 50 generations (Fig. 2). With regard to genetic diversity, average inbreeding levels increased from 0.056 in generation 1 to 0.680 in generation 50, with an inbreeding rate of about 1.5% per generation, while the standard deviation of the underlying true genetic values decreased to 28.9% of the initial population. By generation 50, 64.0% of all SNPs and 74.5% of all QTLs (77.1% of those for the favorable variant) were fixed. Because different metrics for genetic diversity resulted in very similar rankings across scenarios, the following results will focus on genetic gain and inbreeding. Detailed per-generation results on genetic gain, inbreeding levels, genetic variance, and share of QTLs fixed for all considered scenarios are in the supplementary materials [Additional file 1, Table S1 to Additional file 6, Table S6].

### Weighting allele effects by allele frequency

**Fig. 3:**
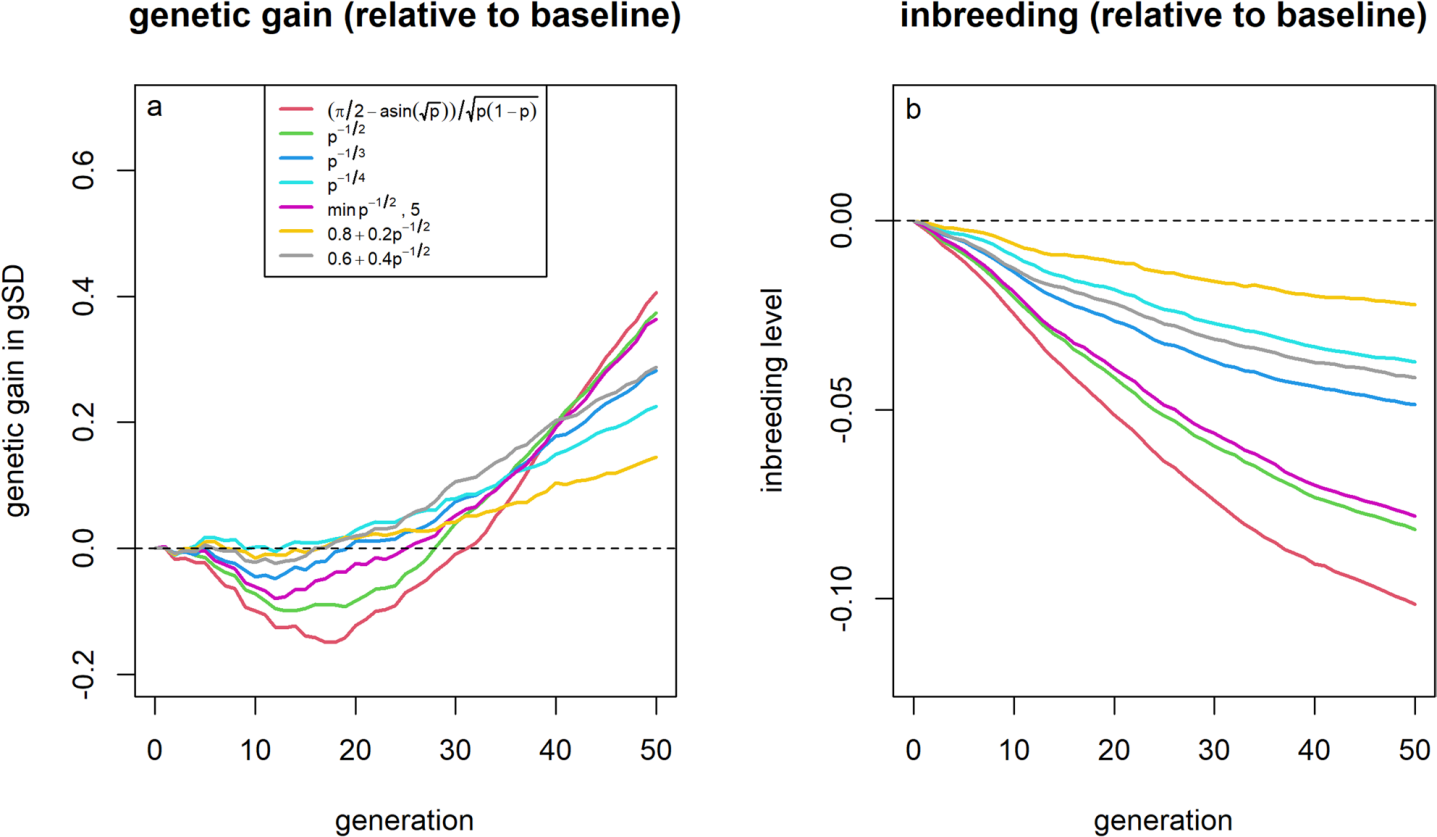
Genetic gain and inbreeding levels in the allele frequency based effect weighting scenarios. Genetic gains (**a**) and inbreeding levels (**b**) relative to selection based on estimated breeding values for different weighting factors for SNP effects depending on the allele frequency of the beneficial variant. Due to the small overall impact of allele weighting strategies and high variance in outcomes, figures were generated based on 500 replicates to reduce variance

The largest long-term genetic gain was observed, when using the theoretical optimal allele weightings proposed by Goddard [20], with a 0.41 gSD (+ 1.6%) greater genetic gain than the baseline scenario, while the average inbreeding level after 50 generations was by 0.102 (− 16.2%) lower than in the baseline scenario (Fig. 3). However, these weightings led to lower short-term genetic gain than the baseline scenario, by up to 0.15 gSD (− 1.2%), with the break-even point for genetic gain reached only after 25 generations. Applying a weaker relative weighting on benefical rare alleles (${p}^{- \frac{1}{4}}$) resulted in almost no additional losses in short-term genetic gain (− 0.01 gSD; − 0.1%), while achieving about half of the additional long-term genetic gain (+ 0.23 gSD; + 0.9%) compared to using weights proposed by Goddard [20] and a notable reduction in inbreeding levels in generation 50 (− 0.037; − 6.0%). Similar results were also obtained when assuming that SNP effects were known [See Additional File 7, Figure S1].

### Relatedness to current breeding population

**Fig. 4:**
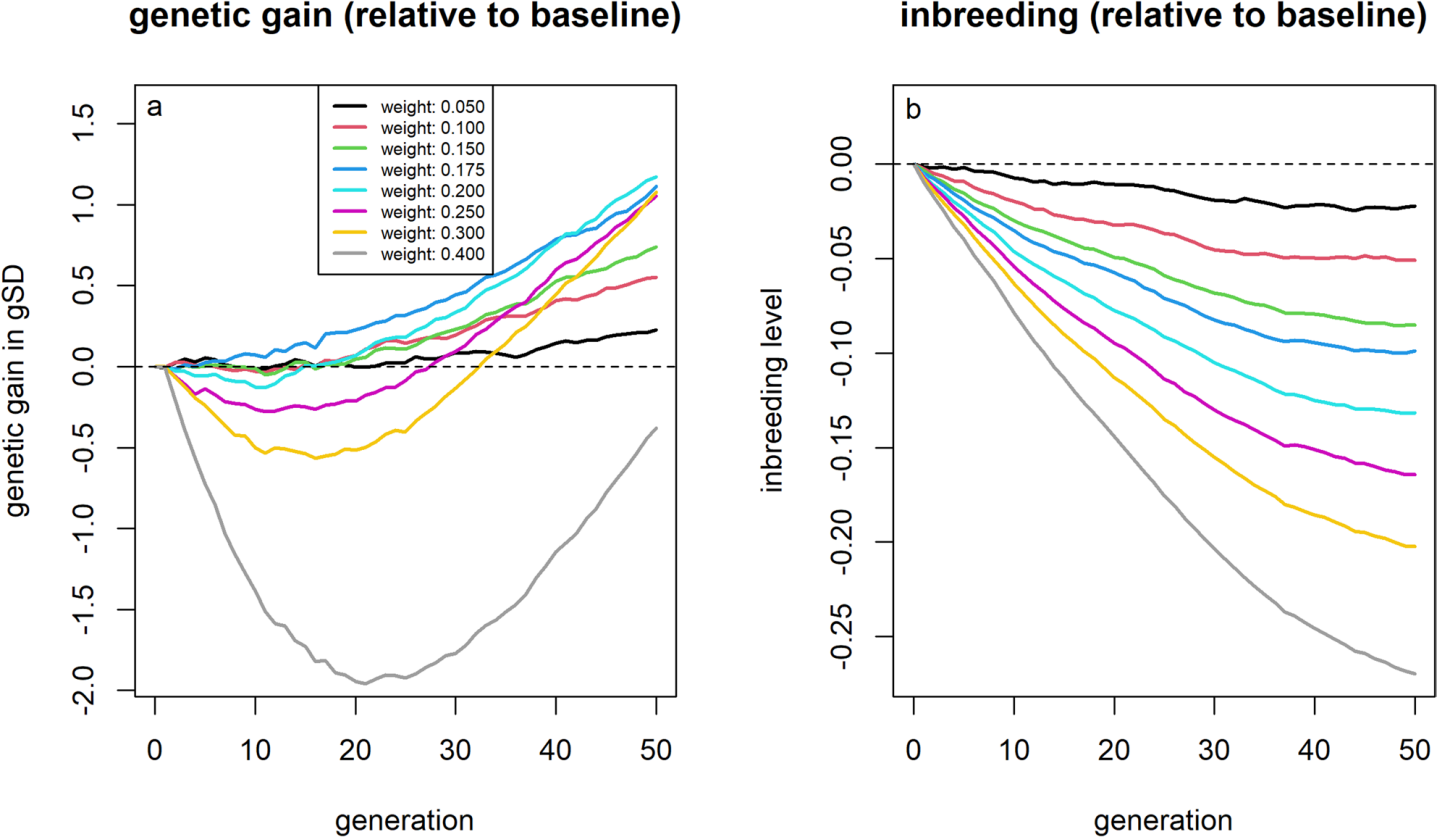
Genetic gain and inbreeding levels for scenarios that included average kinship in the selection index. Genetic gains (**a**) and inbreeding levels (**b**) relative to selection based on estimated breeding values for different index weights for the average kinship of individual to the individuals with the highest estimated breeding values from the current population

When the relative weight assigned to the average kinship of an individual to top individuals in the current breeding cycle was kept below 20%, virtually no short-term losses in genetic gain were observed (Fig. 4). A weighting of 17.5% resulted in a + 1.11 gSD (+ 4.3%) greater genetic gain and 0.099 (− 15.8%) lower inbreeding levels after 50 generations compared to the baseline. In contrast, selection based on the average kinship of an individual to the overall population was less advantageous, yielding lower genetic gain and a smaller reduction in inbreeding levels [See Additional File 8, Figure S2].

### Avoiding matings between related individuals

**Fig. 5:**
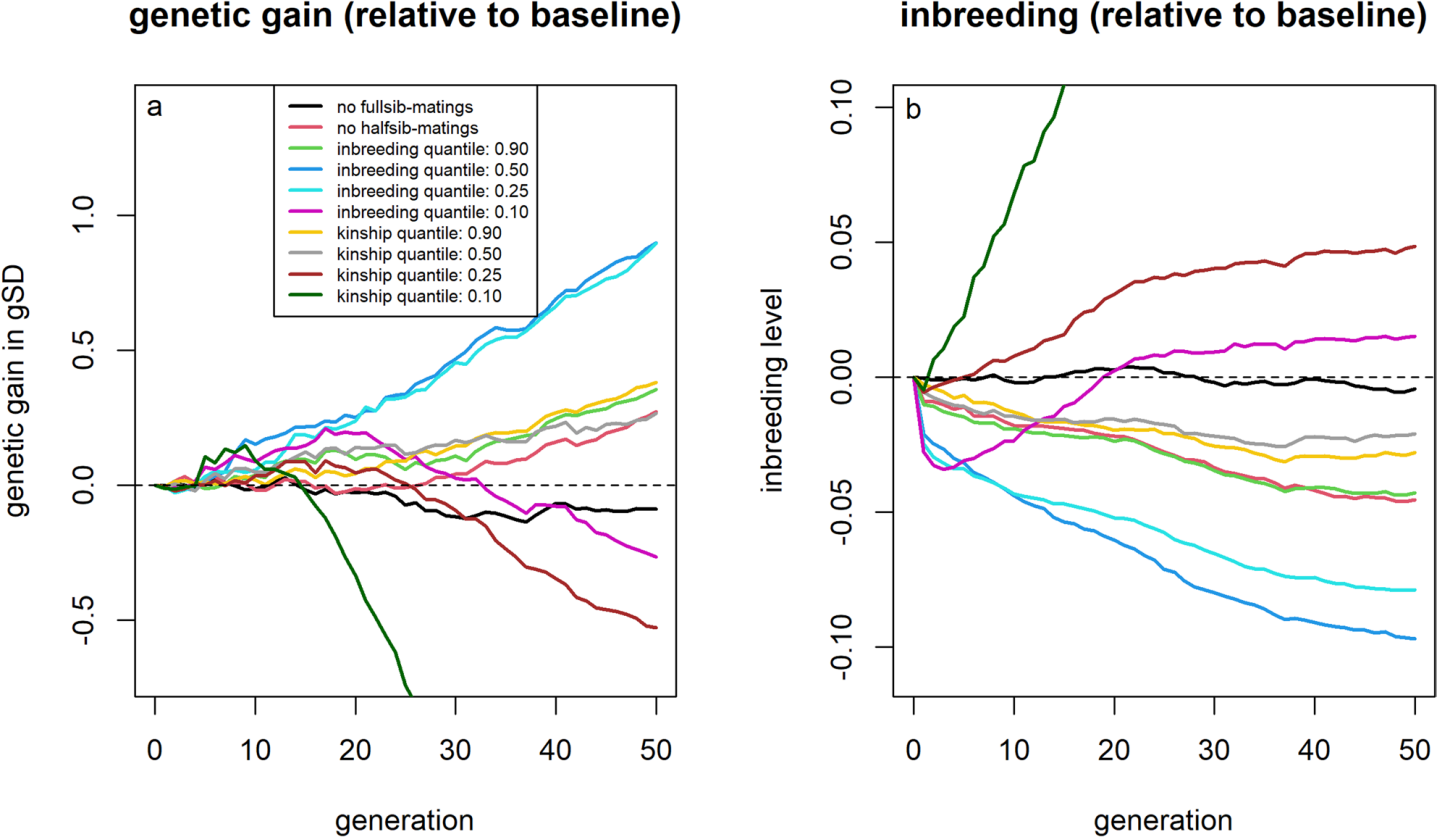
Genetic gain and inbreeding levels for scenarios that included avoiding matings between specific sire-dam combinations. Genetic gains (**a**) and inbreeding levels (**b**) relative to selection based on estimated breeding values for strategies to avoid matings between specific sire-dam combinations

Avoiding matings between full-sibs had no noticeable effect on either genetic gain or inbreeding, as such matings occurred only rarely in the baseline scenario (Fig. 5). Extending this restriction to half-sib matings resulted in lower inbreeding levels (− 0.045 / − 7.3%) and a modest increase in genetic gain (+ 0.27 gSD; + 1.0%) after 50 generations compared to the baseline scenario.

Avoiding matings between individuals that would result in high expected inbreeding levels led to greater improvements. Using the 50%-quantile as a threshold led to additional genetic gains of 0.90 gSD (+ 3.4%) and reduced inbreeding levels by 0.097 (− 15.5%) compared to the baseline scenario. Avoiding matings based on expected kinship of potential offspring to the current breeding population resulted in lower inbreeding levels, however, with a large proportion of matings excluded, the increased use of a limited number of sires and dams led to higher inbreeding levels and lower genetic gain. A detailed comparison across different quantiles for both mating− avoidance strategies is provided in the supplementary material [Additional File 9, Figure S3 and Additional File 10, Figure S4].

### Optimum contribution selection

**Fig. 6:**
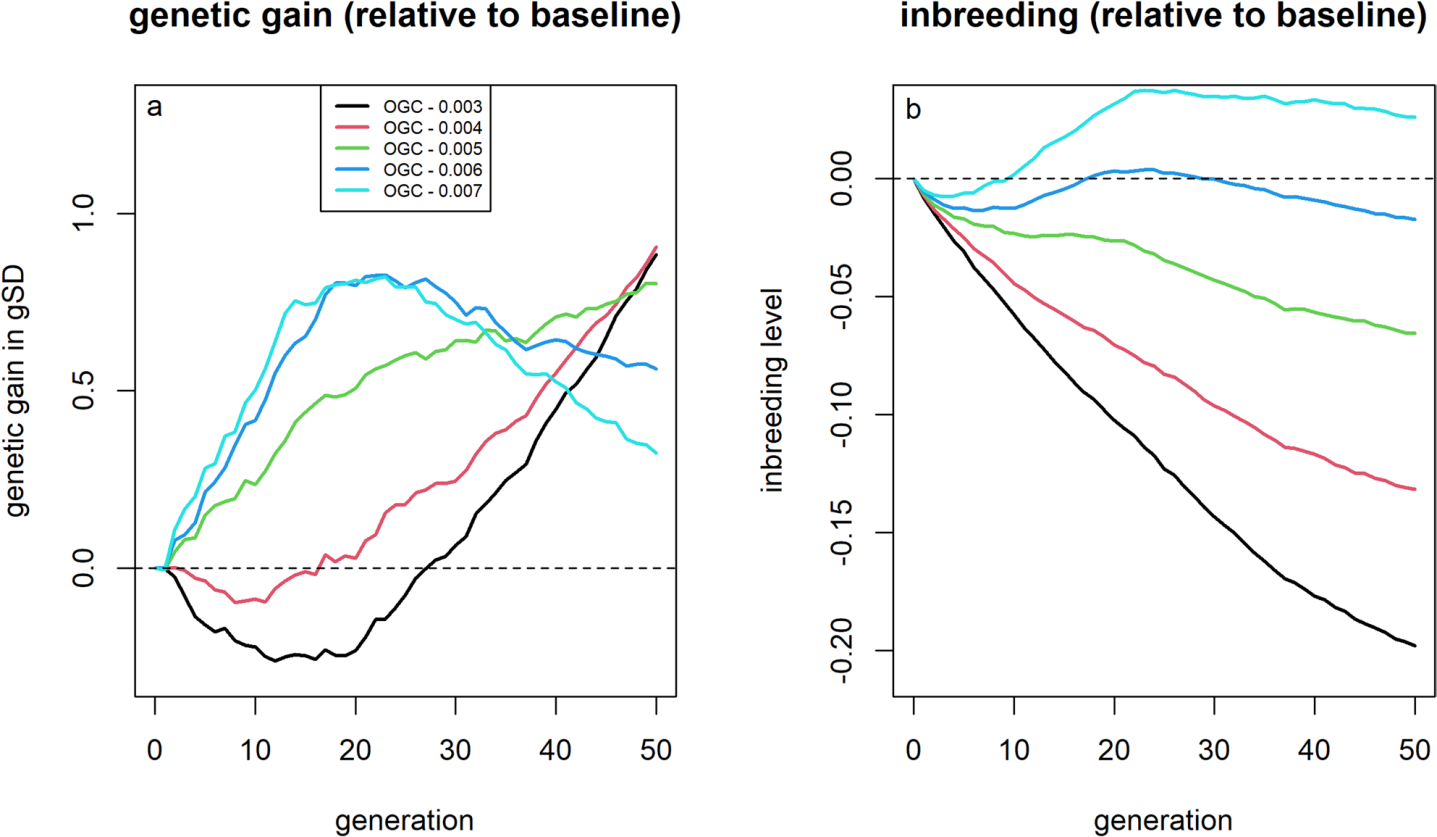
Genetic gain and inbreeding levels for scenarios using optimum contribution selection (OCS) using optiSel with a fixed threshold for the increase in average kinship. Genetic gains (**a**) and inbreeding levels (**b**) relative to selection based on estimated breeding values for different inbreeding constraints when using optimum contribution selection

Applying OCS, solved deterministically through optiSel, with a fixed constraint on inbreeding rates, was more efficient than selection only based on EBVs in the baseline scenario (Fig. 6). Here, we define “efficient” as achieving higher short- and long-term genetic gain along with lower inbreeding levels. When restricting inbreeding rates to at most 0.5% for selection among males, greater genetic gain (+ 0.59 gSD; + 2.3%) and lower inbreeding levels (− 0.062; − 10.0%) were obtained after 50 generations compared to the baseline scenario. Imposing stronger restrictions on the increase in inbreeding led to higher long-term genetic gain and reduced inbreeding but came at the cost of reduced short-term genetic gain.

**Fig. 7:**
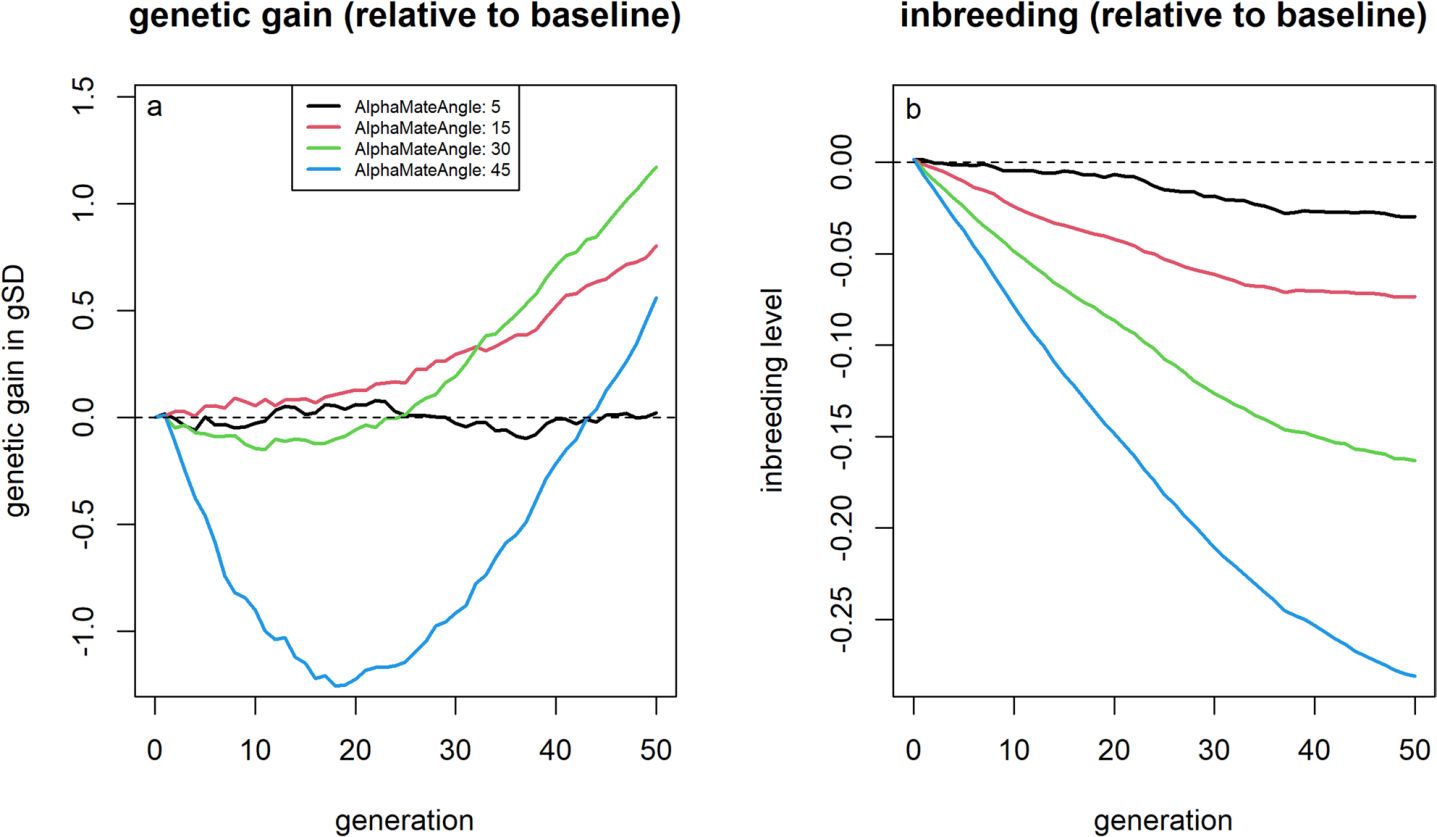
Genetic gain and inbreeding levels for scenarios using AlphaMate, aiming to achieve approximately $\mathrm{cos}\left(\theta \right)$ of the maximal obtainable genetic gain and $\mathrm{sin}(\theta )$ of the minimum obtainable average kinship, depending on the chosen angle $\theta$. Genetic gains (**a**) and inbreeding levels (**b**) relative to selection based on estimated breeding values for different weightings (angles) in AlphaMate between genetic gain and diversity

Solving the OCS problem using AlphaMate, with kinship penalized but without a fixed threshold on increases in average kinship, an angle of 30° led to substantially greater long-term genetic gain (+ 1.17 gSD; + 4.5%) and lower inbreeding level (− 0.163, − 26.2%) than the baseline scenario (Fig. 7). However, short-term genetic gain was reduced by up to 0.15 gSD (− 1.8%) compared to the baseline, with the break-even point for genetic gain reached after approximately 25 generations. Placing greater emphasis on genetic gain (angle = 15°) reduced long-term benefits (genetic gain: + 0.80 gSD; inbreeding level: -0.073) but avoided short-term losses in genetic gain. Further increasing the weight on genetic gain (angle = 5°) did not yield additional improvements in either genetic gain or inbreeding levels.

### Mendelian sampling variance

**Fig. 8:**
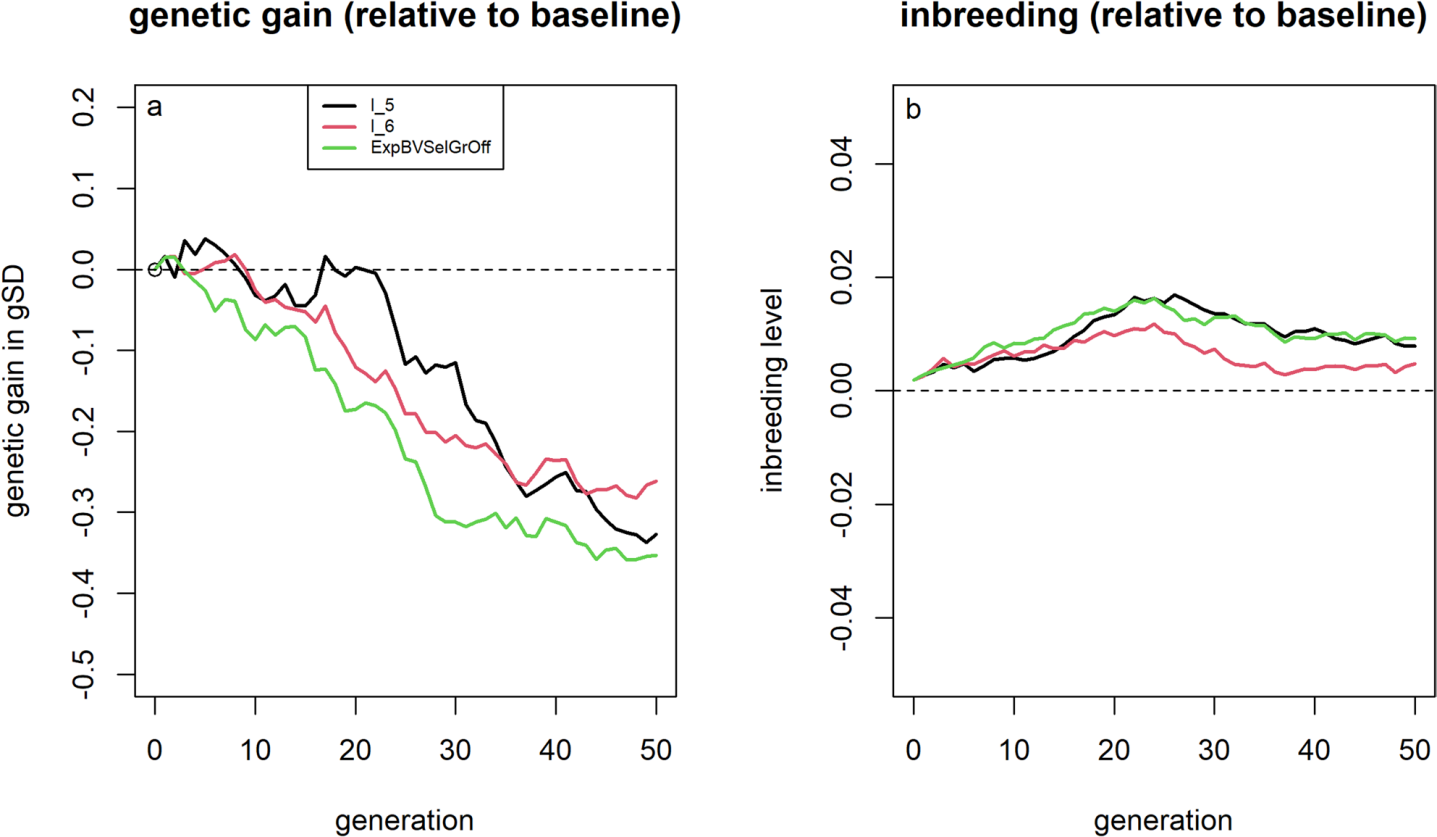
Genetic gain and inbreeding levels for scenarios that accounted for Mendelian sampling variance in selection. Genetic gains (**a**) and inbreeding levels (**b**) relative to selection based on estimated breeding values for different approaches to account for Mendelian sampling variance in selection

All three approaches evaluated for incorporating MSV into selection decisions led to slightly lower genetic gain and marginally higher inbreeding levels compared to selection based only on EBVs (Fig. 8). Across the 50 simulated generations, the average prediction accuracy for MSV was 0.17, markedly lower than the observed prediction accuracy for EBVs (0.53). Both accuracies were calculated as the correlation between the predicted values and the underlying true values. Including individuals from the previous six generations for breeding value estimation improved prediction accuracies to 0.32 for MSV and 0.58 for EBVs. Genetic gain and inbreeding levels in this scenario were comparable to those in the baseline scenario, without yielding additional improvements [See Additional File 11, Figure S5].

### Adapted selection proportions

**Fig. 9:**
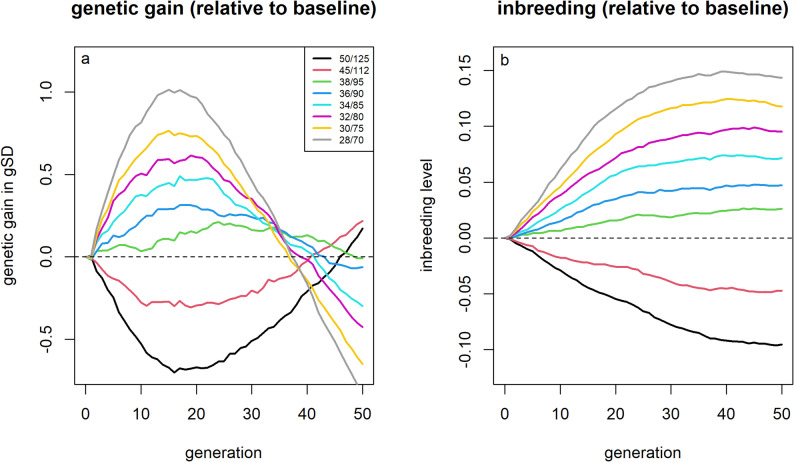
Genetic gain and inbreeding levels for scenarios with different selection proportions. Genetic gains (**a**) and inbreeding levels (**b**) relative to selection based on estimated breeding values depending on the used selection proportion

Reducing the proportion of individuals selected substantially increased short-term genetic gain compared to the baseline scenario (Fig. 9). A 30% reduction in selection proportions increased genetic gain by up to 1.01 gSD (+ 10.0%) after 15 generations. However, this improvement came at the cost of 42% higher inbreeding levels by generation 15. While selecting fewer individuals led to higher genetic gain in the first 20 generations, subsequent genetic gain was reduced, with the break-even point for different selection proportions reached between 35 and 50 generations. In contrast, selecting a larger proportion of individuals yielded smaller short-term gains compared to the baseline scenario and was only marginally advantageous in terms of genetic gain after 50 generations. Across all scenarios, increasing short-term genetic gain by 1% by adjusting selection proportions resulted in an increase in inbreeding rates by 4 to 5%.

### Joint optimization

The evolutionary pipeline used for the joint optimization scenarios does not suggest an optimum per se, which makes the choice of the finally chosen optimum somewhat arbitrary and open to interpretation. However, in both variants considered for this scenario, i.e. the used longer-term versus shorter-term target functions, no substantial improvements in the target function were obtained after iteration 50 of the pipeline, suggesting practical convergence. Suggested optima for each iteration of the evolutionary pipeline and the associated estimated values for the respective target functions are provided in the supplementary materials [Additional File 12, Figure S6 and Additional File 13, Figure S7].

For both variants of the target function, the pipeline consistently suggested the use of OCS solved with optiSel, combined with a moderate weighting of relatedness to top individuals in the selection index (17 and 9% for the respective variants). Furthermore, matings between related individuals were avoided, excluding 20 and 45% of all potential matings based on expected inbreeding for the two respective target functions, while only 10% of matings were avoided based on kinship for both variants (Table 2).

**Table 2:** Suggested breeding program designs by the joint optimization for the two variants of the target function

Parameter	Longer-term target function	Shorter-term target function
Weighting factor for the beneficial alleles $= \frac{\frac{\pi }{2}-\mathrm{arcsin}\left(\sqrt{p}\right)}{\sqrt{\left(1-p\right)\cdot p}}$	0.00	0.20
Weighting factor for the beneficial alleles $={p}^{-\frac{1}{3}}$	1.00	0.82
Weight on average relatedness to top individuals	0.17	0.09
Weight on average relatedness to breeding population	0.00	0.02
Number of selected females	83	32
Quantile to avoid matings based on expected inbreeding	0.80	0.55
Quantile to avoid matings based on expected kinship	0.90	0.90
Increase of average kinship in optimal contribution selection	0.005	0.012
Binary on whether deterministic optimal contribution selection is used?	TRUE	TRUE

The evolutionary pipeline further suggested placing strong emphasis on upweighting beneficial rare alleles using the moderate weighting of ${p}^{-\frac{1}{3}}$, while only limited weight was put on the theoretically optimal weighting suggested by Goddard [20]. For both variants of the target function, basically no weight was placed on average relatedness of an individual to the overall breeding population. The primary difference between the optimized breeding schemes for the two variants was a much lower proportion of selected individuals for the variant with more focus on short-term gain, selecting 83 and 32 dams, respectively (compared to 100 in the baseline).

**Fig. 10:**
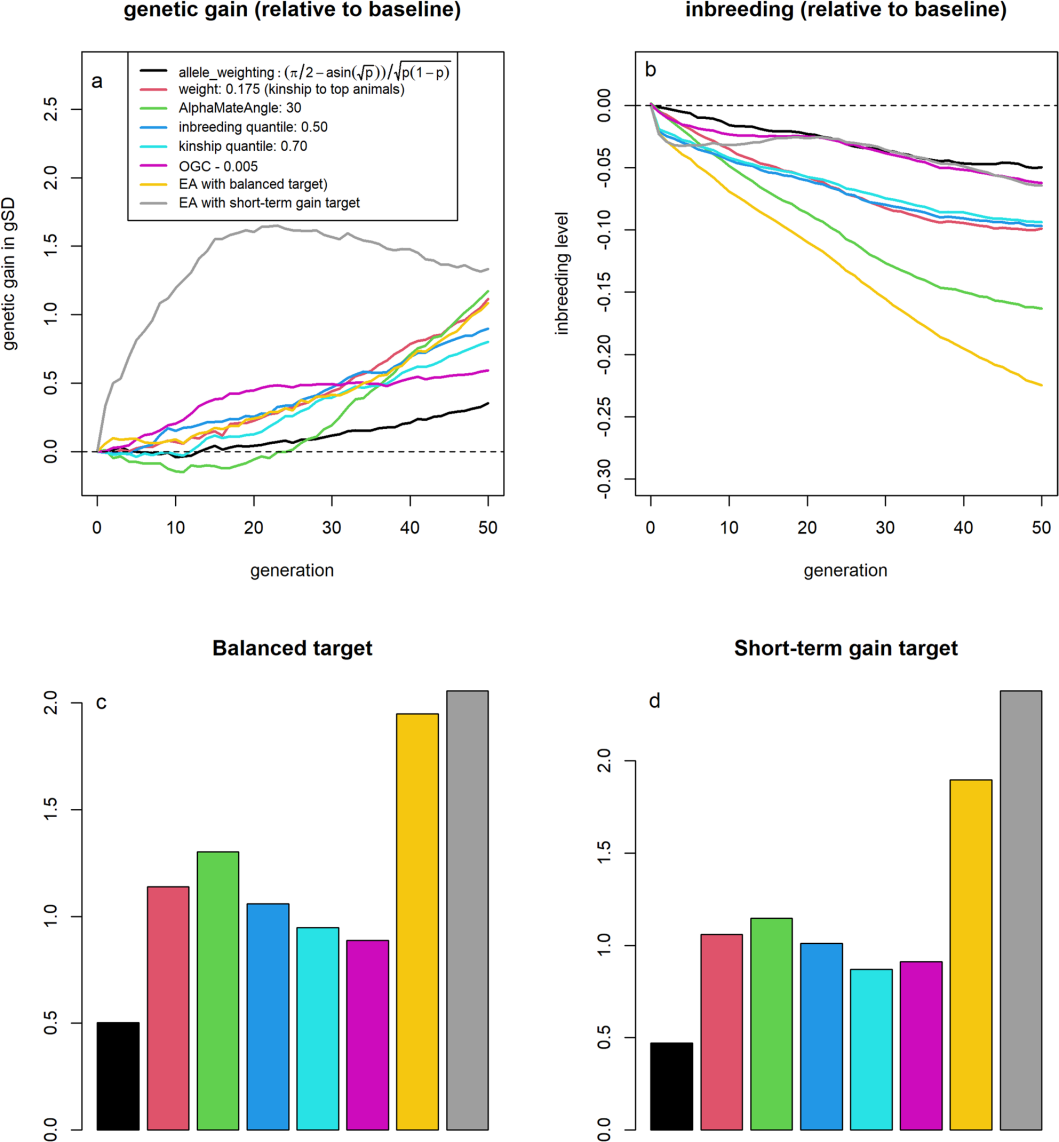
Genetic gain and inbreeding levels for scenarios of joint strategies optimized using the evolutionary algorithm. Genetic gain (**a**) and inbreeding levels (**b**) for various diversity management strategies relative to selection based on estimated breeding values. All scenarios are additionally evaluated on their performance on longer-term target function (**c**) and the target function with focus shorter-term genetic gain (**d**) relative to selection based on estimated breeding values

The breeding program optimized using the target function with focus on longer-term genetic gain led to an increase in genetic gain of 1.34 gSD (+ 5.1%) and a reduction in inbreeding levels by 0.232 (− 37.3%) after 50 generations compared to the baseline (Fig. 10a and b). Reductions in levels of inbreeding were obtained consistently across all generations, whereas genetic gain was only on par with the baseline scenario for the first 20 generations. In contrast, the breeding program optimized using the target function with focus on shorter-term genetic gain resulted in a more modest reduction of inbreeding levels by 0.064 (− 10.3%) compared to the baseline scenario but achieved comparable long-term genetic gains of 1.33 gSD (+ 5.1%). Although a reduction in the level of inbreeding was again obtained across all generations, additional genetic gain was primarily obtained in the first 20 generations, with an additional genetic gain of 0.33 gSD in the first generation (+ 31.1%).

In comparison to breeding program designs that only considered a single management strategy, joint application of all strategies led to much better performance for the target function considered (Fig. 10c and d). The best single management strategy resulted in an improvement in the longer-term target function by 1.15 units and 1.30 in the shorter-term target function relative to the baseline scenario, while the joint strategy led to an increase of the target function by, respectively, 1.96 and 2.37. Both breeding schemes suggested by the joint optimization pipeline achieved higher genetic gain than other strategies that resulted in similar inbreeding rates per generation, indicating improved efficiency.

## Discussion

This study provides an extensive comparison of potential selection strategies for management of genetic diversity and inbreeding in a breeding program, evaluated using stochastic simulations. Rather than proposing new selection strategies, the primary focus of this study was on systematically assessing previously proposed approaches and combinations thereof, within a unifying testing framework. Our results indicate major differences in outcomes of a breeding program depending on the selection strategy used. These findings highlight the need for careful assessment of different approaches to ensure efficient breeding program design, particularly for breeding companies that must balance short-term genetic gain and commercial performance while maintaining a sustainable and genetically diverse breeding population [41].

### Individual selection strategies

Increasing the weight of SNP effects for loci with beneficial rare alleles using theoretical optimal long-term weights [20] only yielded higher genetic gain compared to strict selection on EBV after 30 generations, which for dairy cattle corresponds to a time horizon of 75 years, rendering this approach impractical for most commercial breeding programs [42]. In contrast, more moderate upweighting of beneficial rare alleles (${p}^{- \frac{1}{3}}$) also yielded higher long-term genetic gain than strict selection based on EBVs, while avoiding short-term losses in genetic gain, making it more feasible for implementation in a commercial breeding program.

Overall, weighting SNP effects based on allele frequencies had a comparatively modest impact compared to other approaches evaluated. Although the breeding population and the training population used for genomic prediction were relatively small, we do not expect substantial differences in larger populations, as the use of the actual QTL effects and genotypes for selection and allele weighting led to similar results, including short-term losses and long-term benefits [See Additional File 7, Figure S1].

Including the average kinship of selection candidates to top individuals in the population based on EBVs as an additional trait in the selection index performed similar to OCS [17]. For example, an index weight of 0.175 on average kinship led to very similar genetic gain and inbreeding to the use of OCS with inbreeding rate limited to 0.43% per generation on the male side [See Additional File 14, Figure S8], indicating very similar efficiency of these two approaches. From a practical perspective, this approach offers clear advantages over OCS, as it relies on a simple numeric score for each selection candidate, is computationally less demanding, and is easier to implement in routine breeding decisions. However, unlike OCS, selection on average kinship does not allow for substantially increasing short-term gain by relaxing the inbreeding constraint and would instead require reducing selection proportions.

In contrast to the previous studies [26, 28, 30], we did not observe benefits from accounting for predicted progeny variance based on MSV in selection, which may be because the breeding program design considered here was not suitable for the application of such strategies. First, selection proportions in our breeding scheme were relatively high (8% for males and 20% for females), whereas Bijma et al. [25] suggested that accounting for MSV will primarily be effective with selection proportions of 5% or less. Second, assortative mating strategies, which have been shown to enhance the effectiveness of selection on MSV [26, 27, 30], were not tested in this study. Third, the prediction accuracy for MSV was low in our study (0.17). Expanding the reference population improved prediction accuracies (0.30) and resulted in inbreeding levels and genetic gain comparable to those achieved with strict selection on EBVs [See Additional File 11, Figure S5].

Although not traditionally considered a progeny variance approach, avoiding mating between related individuals can conceptually promote a higher probability of generating novel haplotypes and higher variation of potential offspring, without the noise introduced by inaccurate estimates of SNP effects. The results here highlight very limited downsides of avoiding matings between individuals that have high pairwise kinships and, therefore, high expected inbreeding levels of their offspring.

From a practical breeding perspective, most of the evaluated strategies reduced inbreeding rates but at the expense of having lower short-term genetic gain. However, these short-term losses can be offset by reducing selection proportions and/or applying a weaker inbreeding constraint in OCS. Notably, an increase in short-term genetic gain of 1% by changing selection proportions led to an increase in inbreeding rates by 4 to 5%. Therefore, a ratio between genetic gain and inbreeding, as for example provided in [43, 44], should not be used as the sole metric to assess long-term efficiency of a breeding program, as it will inherently favour strategies with lower genetic gain and inbreeding over a fixed time horizon. Instead, scenarios can be made more comparable by adjusting selection proportions to achieve similar inbreeding rates. This principle is illustrated in this work by the comparison between OCS and using average kinship to top individuals as part of the selection index. Therefore, selection strategies with lower short-term genetic gain than selection based on EBVs can still be more efficient when they substantially reduce inbreeding rates. Particularly for species with shorter generation intervals, such as black soldier flies, the ratio between genetic gain and inbreeding rate remains a useful complementary metric [45], as such breeding schemes can rapidly approach states of depleted genetic diversity and limited potential for further genetic progress.

### Joint optimization

The breeding program designs suggested by the joint optimization pipeline based on an evolutionary algorithm [34] demonstrate that maximizing the efficiency of selection decisions in breeding program design requires combining multiple approaches for managing genetic diversity, rather than relying on a single strategy. Although the results of the joint optimization pipeline were strongly influenced by the chosen target function, they nevertheless allow for robust conclusions regarding which selection strategies are most effective and how different strategies complement one another. While weighting SNP effects by allele frequency of the beneficial allele had only a modest standalone impact, the results suggest that it acted complementary to OCS. Consequently, a moderate upweighting of beneficial rare alleles (${p}^{-\frac{1}{3}}$) was included with substantial weighting in the optimum strategy for both target functions evaluated. Notably, despite the substantially lower selection proportions recommended by the joint optimization pipeline, the resulting inbreeding levels were lower than in the baseline scenario with selection based on EBVs.

The optima identified by the joint optimization pipeline [34] should not be interpreted as globally optimal solutions, but rather as highly suitable breeding programs within the explored search space. Notice that the proposed breeding program design based on the shorter-term target function had a higher value for the target function of the longer-term target function than the optimum that was obtained from the evolutionary algorithm for that target function (Fig. 10c). Potential reasons for this are that the global optimum is outside the initial search space, rendering it difficult to reach through the applied optimization procedure. Furthermore, the optimization pipeline employs local smoothing, which may disproportionally penalize breeding program designs with small selection proportions, as minor deviations of design parameters will impact absolute outcomes more [34]. Despite these limitations, the breeding program designs proposed by the evolutionary algorithm substantially outperformed all selection strategies based on a single selection strategy, underscoring the utility of the evolutionary algorithm as a powerful tool for improving breeding program design.

From a practical perspective, two major challenges limit the use of the evolutionary algorithm for such optimization problems: First, in the evolutionary algorithm, 100 iterations with a total of 30,700 simulations were performed, which for larger breeding schemes can require substantial computational costs and requires highly efficient simulation software [8, 10]. One potential solution for this could be to simplify the breeding program in a manner that affects all considered scenarios in a similar way, e.g., to reduce the computing time of breeding value estimation by reducing the number of traits or generations considered. Note that, for computational reasons, the use of AlphaMate and MSV-based selection were not included in the optimization pipeline. Second, optimization requires specification of an explicit target function. While assigning economic weights to traits can already be challenging in practise, we expect that defining appropriate weights for inbreeding rates and genetic diversity is even more difficult. A pragmatic solution for this could be to explore multiple target functions and subsequently evaluate the resulting optima in terms of their expected genetic gains and inbreeding. This comparative analysis can then inform the selection of the breeding program design that is the most suitable for implementation.

Note that in the optimization problem considered, a constant breeding program design was used across all 50 generations. In practice, if the breeding objective is to maximize genetic gain at a predefined time point rather than to achieve continuous improvement of the breeding population, further improvements may be attainable by allowing the breeding program designs to change over generations. However, introducing time-dependent decision variables would substantially increase the number of parameters and simulations required. In such cases, AI-based optimization approaches such as reinforcement learning may be more suitable [46].

### Practical considerations and implementation

Although the final optimum identified by the evolutionary algorithm incorporated a combination of multiple selection strategies, we acknowledge that, from a practical perspective, simpler models may be preferable if they capture most of the benefits while being easier to implement and maintain. The results for the individual strategies provide guidance on which approaches are most impactful, with the use of MSV and weighting of SNP effects based on allele frequency being candidates for exclusion, as their overall impact was low. Furthermore, the evolutionary optimization pipeline could be adapted to reduce complexity, either by limiting the number of parameters or by including a penalty in the target function for incorporating additional selection strategies.

As the breeding program considered in this study was chosen to be generic (with the strongest assumption being that all selection candidates were phenotyped and genotyped), the impact of individual selection strategies we observed is expected to allow for broadly applicable general conclusions regarding their impact in a breeding program. These results can therefore allow for a pre-selection of methods to consider in practice. For subsequent fine-tuning of strategy-specific weightings, stochastic simulation of a breeding program that more closely resembles the real-world breeding program (“digital twin”) can further improve implementation.

Note that the trait considered in this study was assumed to be additive and highly polygenic, without inbreeding depression [47], deleterious variants as dominant QTLs or epistatic interactions [48, 49]. Hence, although inbreeding levels increased over time, genetic gain was only affected by the depletion of genetic variance and not by inbreeding depression per se. As a result, the reported outcomes should be seen as a conservative estimate of the potential benefits of diversity management in breeding programs.

Note that genetic variance in stochastic simulations tends to decrease more rapidly than observed in reality, although similar trends of decreasing heritabilities have been reported in real-world breeding programs in recent years [50–52]. Potential reasons for this include that breeding programs are executed without error in simulations and that trait definitions, breeding goals, and environments may change over time in practise [53–55]. Therefore, our results for the absolute levels of long-term genetic gain and genetic variance should be interpreted with caution. Instead, conclusions should mainly be based on the relative ranking of scenarios. Here, it is essential to critically assess whether different scenarios are similarly affected by the limitations and simplifications of the simulation design and assumptions.

Our analysis by no means has claims of completeness and focused primarily on the selection step itself, rather than on a more fundamental restructuring of the breeding program design by changing generation intervals [16], splitting the breeding scheme into multiple distinct components [15], or making use of cryo reserves [14] or outcross individuals [13]. All simulation and analysis scripts are provided in the supplementary material [See Additional File 15, File S1 and Additional File 16, File S2]. The modular structure and flexibility of the simulation software MoBPS [8] allow for the extension of scripts to include additional selection strategies as well as more general conceptual changes of breeding program design.

## Conclusions

The use of selection strategies for management of genetic diversity and inbreeding does not necessarily lead to short-term losses in genetic gain and can, in fact, be combined with selecting fewer individuals without compromising or even greater genetic gain. The results in this study suggest that the greatest efficiency is achieved through the combination of management and selection strategies, including OCS, weighting of SNP effects based on allele frequency, including average kinship to top individuals as an additional trait in the selection index, avoiding mating between related individuals, and offsetting small short-term losses by selecting fewer individuals. As all considered selection strategies incur no additional costs beyond increased computational effort, their implementation represents a practical and effective pathway for breeding companies to secure long-term genetic progress and population sustainability.

## Supplementary Information

Supplementary material 1 Genetic gain for all considered scenarios per generation.

Supplementary material 2 Inbreeding levels for all considered scenarios per generation.

Supplementary material 3 Remaining genetic variation compared to the baseline for all considered scenario per generation.

Supplementary material 4 Share of SNPs fixated for all considered scenarios per generation.

Supplementary material 5 Share of QTLs fixated for all considered scenarios per generation.

Supplementary material 6 Share of QTLs fixated for the beneficial allele for all considered scenarios per generation.

Supplementary material 7 Genetic gain and inbreeding levels in the allele frequency based effect weighting scenarios. Genetic gains (a) and inbreeding levels (b) relative to selection based on underlying true genetic values for different weighting factors for SNP effects depending on the allele frequency of the beneficial variant, assuming known SNP effects.

Supplementary material 8 Genetic gain and inbreeding levels of the scenarios including average kinship in the selection index. Genetic gains (a) and inbreeding levels (b) relative to selection based on estimated breeding values for different index weights for the average kinship of an individual to the current breeding population.

Supplementary material 9 Genetic gain and inbreeding levels of the scenarios including avoiding matings based on expected inbreeding. Genetic gains (a) and inbreeding levels (b) relative to selection based on estimated breeding values when avoiding different shares of matings between individuals based on the expected inbreeding level of a hypothetical offspring.

Supplementary material 10 Genetic gain and inbreeding levels of the scenarios including avoiding matings based on expected kinship. Genetic gains (a) and inbreeding levels (b) relative to selection based on estimated breeding values when avoiding different shares of matings between individuals based on the expected average kinship level to the current population of a hypothetical offspring.

Supplementary material 11 Genetic gain and inbreeding levels of the scenarios using Mendelian sampling variance in selection. Genetic gains (a) and inbreeding levels (b) relative to selection based on estimated breeding values for different approaches to account for Mendelian sampling variance in selection when including the last six generations in the breeding value estimation.

Supplementary material 12 Results of the evolutionary algorithm with a long-term target function. Optima suggested by the evolutionary algorithm with long-term target function for each iterations for each individual parameter and the estimated value of the target function in the optima. Black dashed lines indicate the initial sampling range per parameter.

Supplementary material 13 Results of the evolutionary algorithm target function with focus on short-term genetic gain. Optima suggested by the evolutionary algorithm with target function with focus on short-term genetic gain for each iterations for each individual parameter and the estimated value of the target function in the optima. Black dashed lines indicate the initial sampling range per parameter.

Supplementary material 14 Comparison of genetic gains and inbreeding levels between optimum contribution selection and kinship in selection. Genetic gains (a) and inbreeding levels (b) relative to selection based on estimated breeding values when using optimum contribution selection with an maximum inbreeding of 0.43% compared to the use of average kinship to top individuals as a trait in the selection index with a weight of 17.5%.

Supplementary material 15 Script used for the simulation of the different breeding program design using the R-package MoBPS.

Supplementary material 16 Script used for analysis of outcomes of the simulations and subsequent generation of plots.

## Data Availability

The software MoBPS and the evolutionary pipeline can be found in the following two GitHub repositories: https://github.com/tpook92/MoBPS and https://github.com/AHassanpour88/Evolutionary_Snakemake. Data sharing is not applicable to this article as no datasets were generated or analysed during the current study.
