# Supplementary figures and images for: Strategies to improve on selection based on estimated breeding values
Source: Genet Sel Evol. 2026 Feb 14;58:13. doi: 10.1186/s12711-026-01034-z (PMC12922190; doi:10.1186/s12711-026-01034-z)

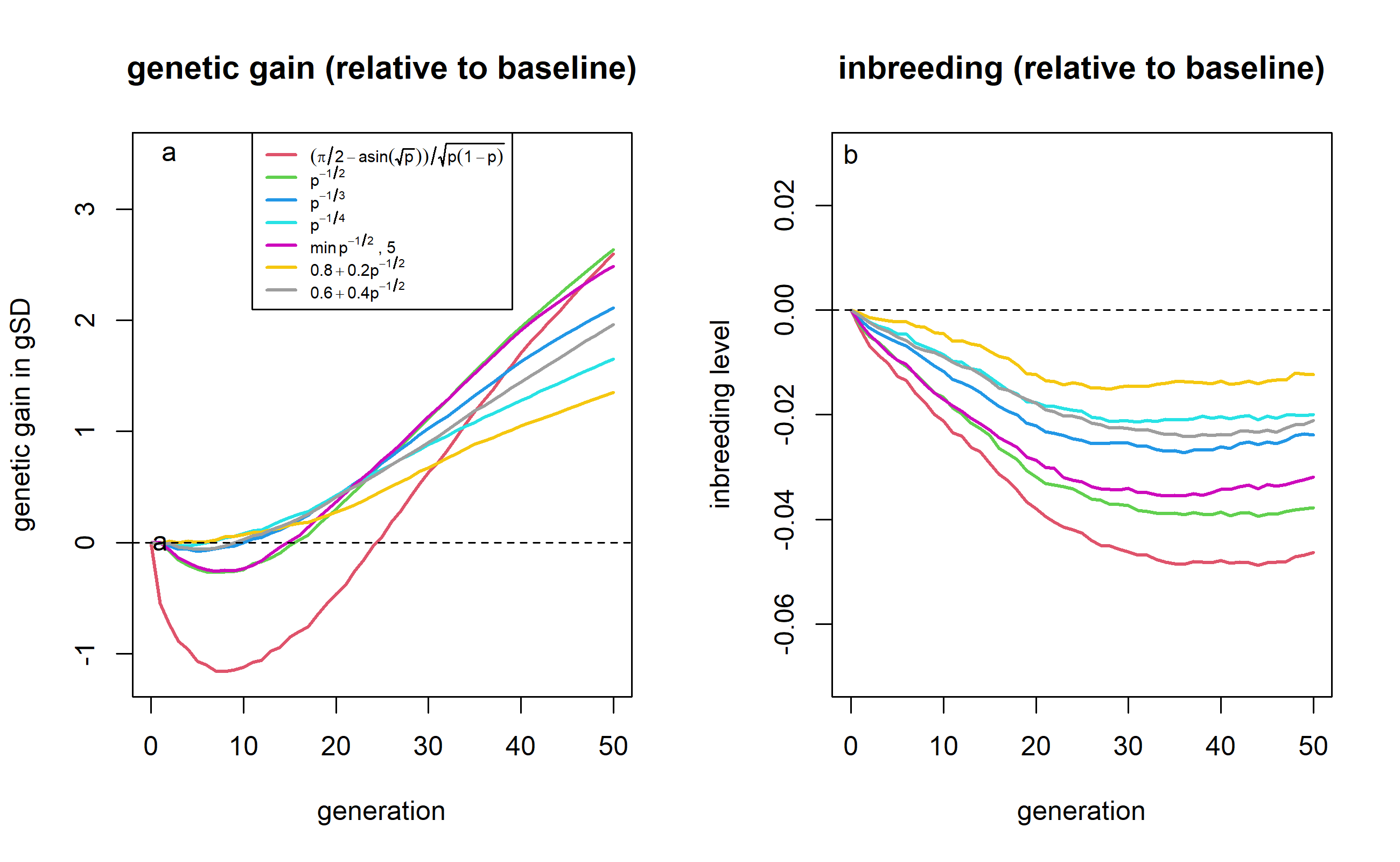

Supplement: Supplementary file 7 — Supplementary material 7 Genetic gain and inbreeding levels in the allele frequency based effect weighting scenarios. Genetic gains (a) and inbreeding levels (b) relative to selection based on underlying true genetic values for different weighting factors for SNP effects depending on the allele frequency of the beneficial variant, assuming known SNP effects. [file 12711_2026_1034_MOESM7_ESM.png]

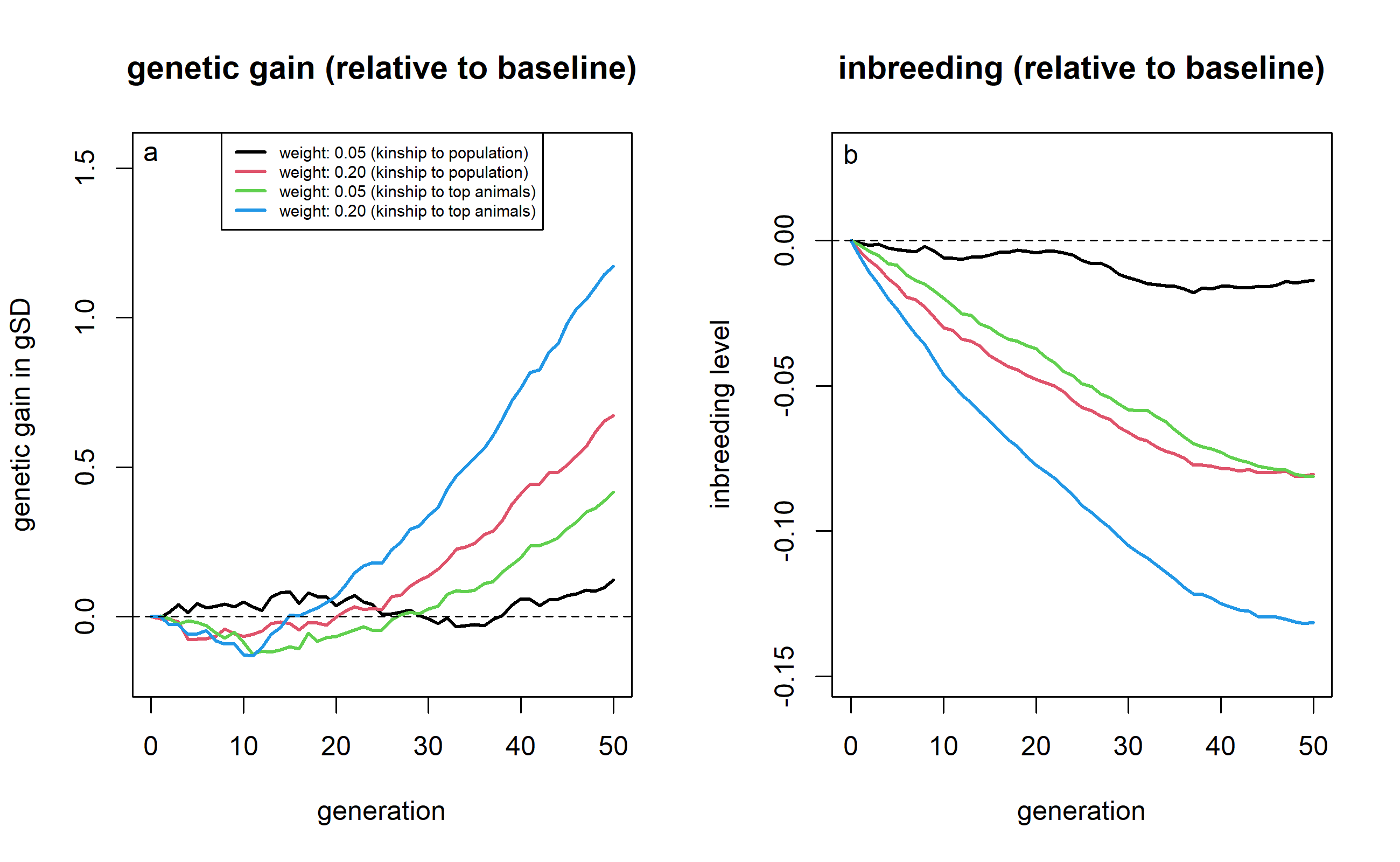

Supplement: Supplementary file 8 — Supplementary material 8 Genetic gain and inbreeding levels of the scenarios including average kinship in the selection index. Genetic gains (a) and inbreeding levels (b) relative to selection based on estimated breeding values for different index weights for the average kinship of an individual to the current breeding population. [file 12711_2026_1034_MOESM8_ESM.png]

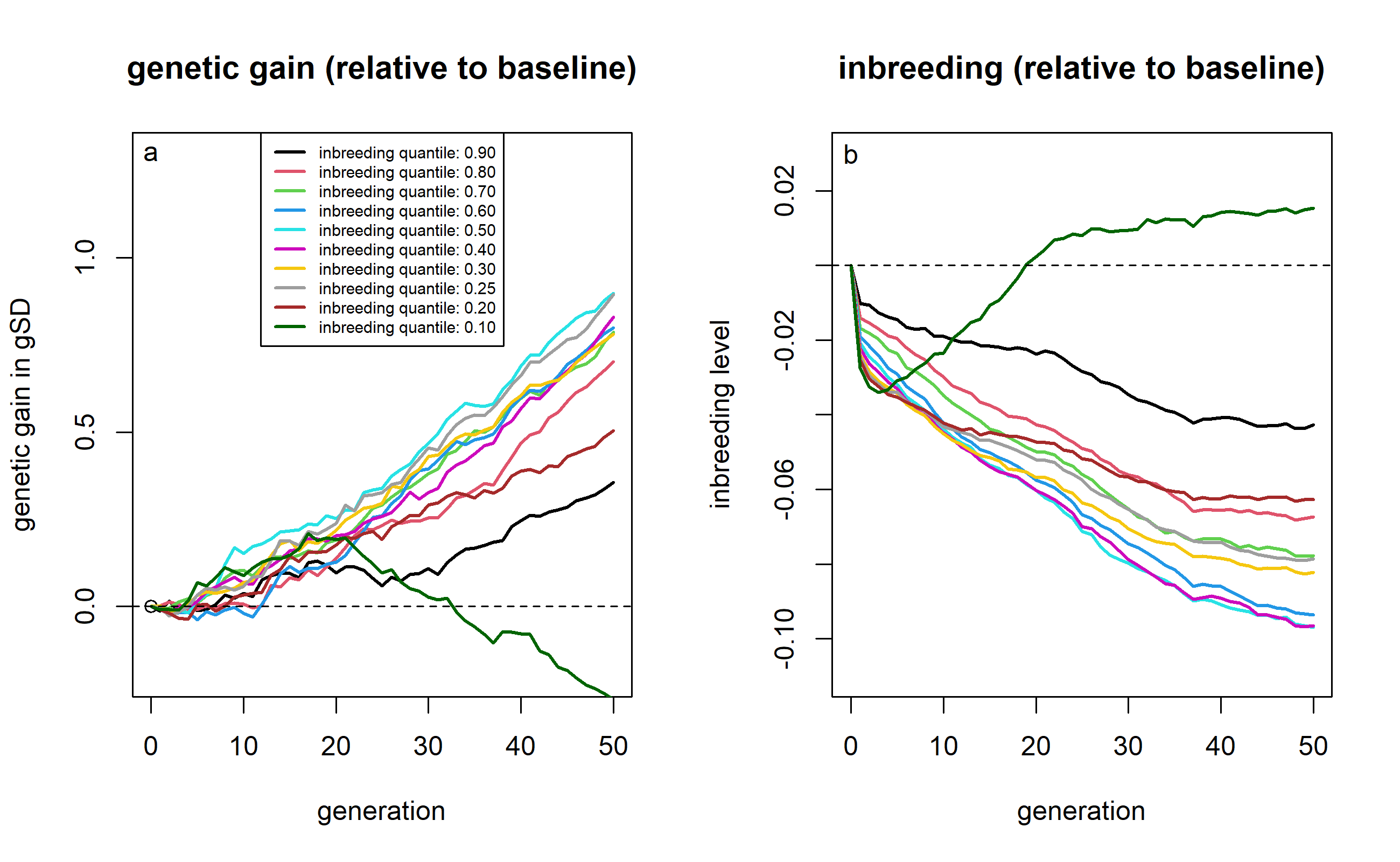

Supplement: Supplementary file 9 — Supplementary material 9 Genetic gain and inbreeding levels of the scenarios including avoiding matings based on expected inbreeding. Genetic gains (a) and inbreeding levels (b) relative to selection based on estimated breeding values when avoiding different shares of matings between individuals based on the expected inbreeding level of a hypothetical offspring. [file 12711_2026_1034_MOESM9_ESM.png]

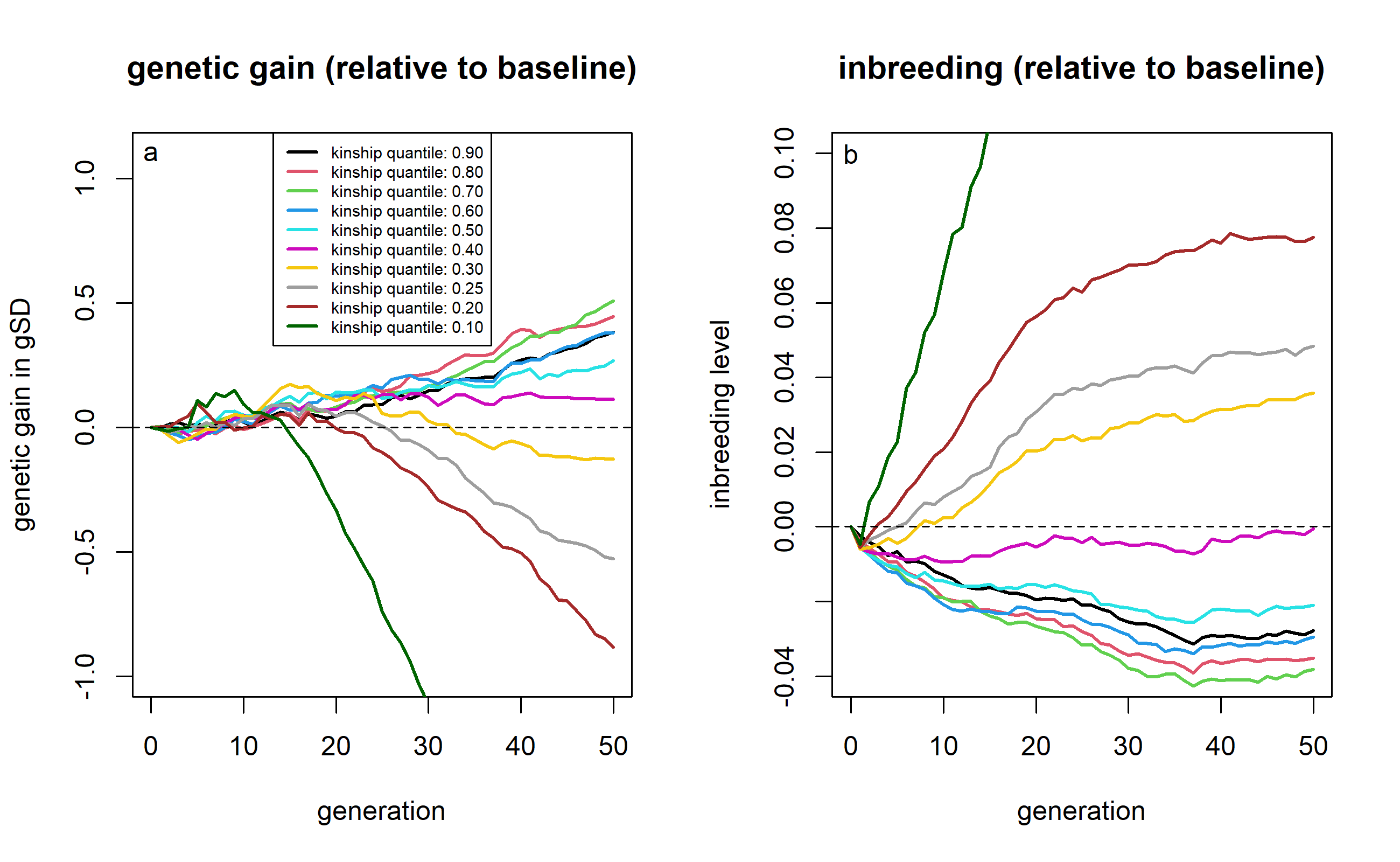

Supplement: Supplementary file 10 — Supplementary material 10 Genetic gain and inbreeding levels of the scenarios including avoiding matings based on expected kinship. Genetic gains (a) and inbreeding levels (b) relative to selection based on estimated breeding values when avoiding different shares of matings between individuals based on the expected average kinship level to the current population of a hypothetical offspring. [file 12711_2026_1034_MOESM10_ESM.png]

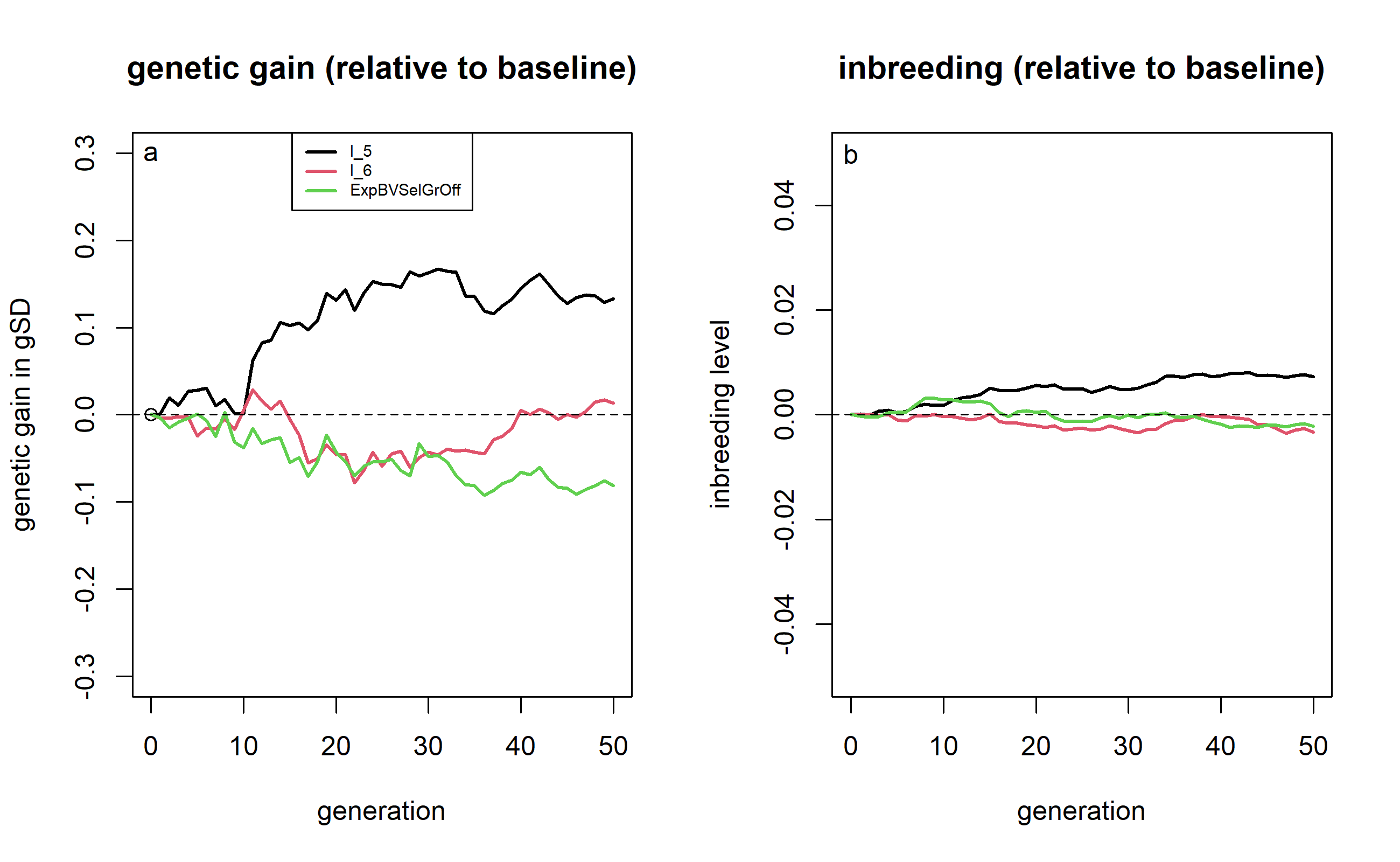

Supplement: Supplementary file 11 — Supplementary material 11 Genetic gain and inbreeding levels of the scenarios using Mendelian sampling variance in selection. Genetic gains (a) and inbreeding levels (b) relative to selection based on estimated breeding values for different approaches to account for Mendelian sampling variance in selection when including the last six generations in the breeding value estimation. [file 12711_2026_1034_MOESM11_ESM.png]

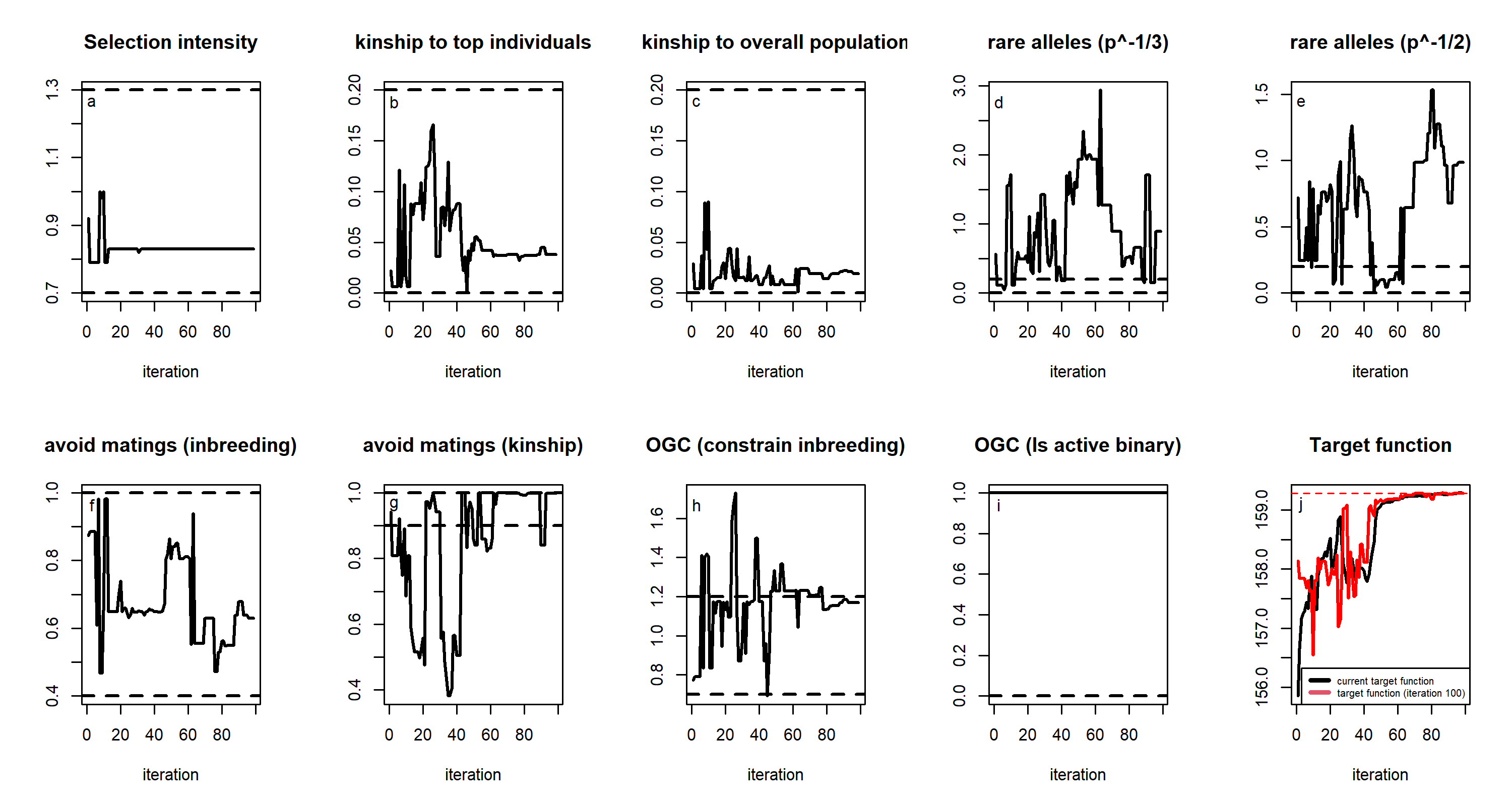

Supplement: Supplementary file 12 — Supplementary material 12 Results of the evolutionary algorithm with a long-term target function. Optima suggested by the evolutionary algorithm with long-term target function for each iterations for each individual parameter and the estimated value of the target function in the optima. Black dashed lines indicate the initial sampling range per parameter. [file 12711_2026_1034_MOESM12_ESM.png]

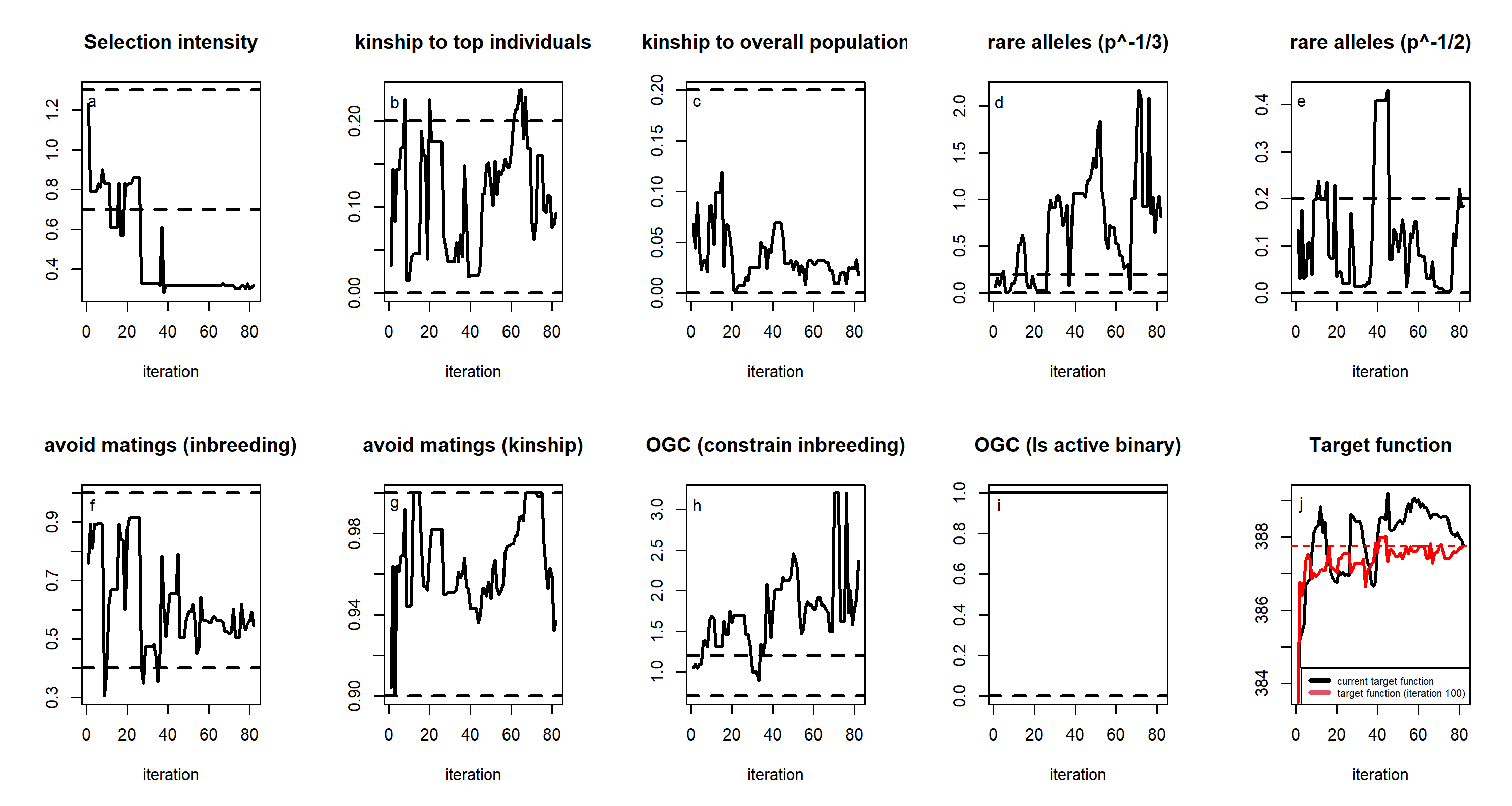

Supplement: Supplementary file 13 — Supplementary material 13 Results of the evolutionary algorithm target function with focus on short-term genetic gain. Optima suggested by the evolutionary algorithm with target function with focus on short-term genetic gain for each iterations for each individual parameter and the estimated value of the target function in the optima. Black dashed lines indicate the initial sampling range per parameter. [file 12711_2026_1034_MOESM13_ESM.png]

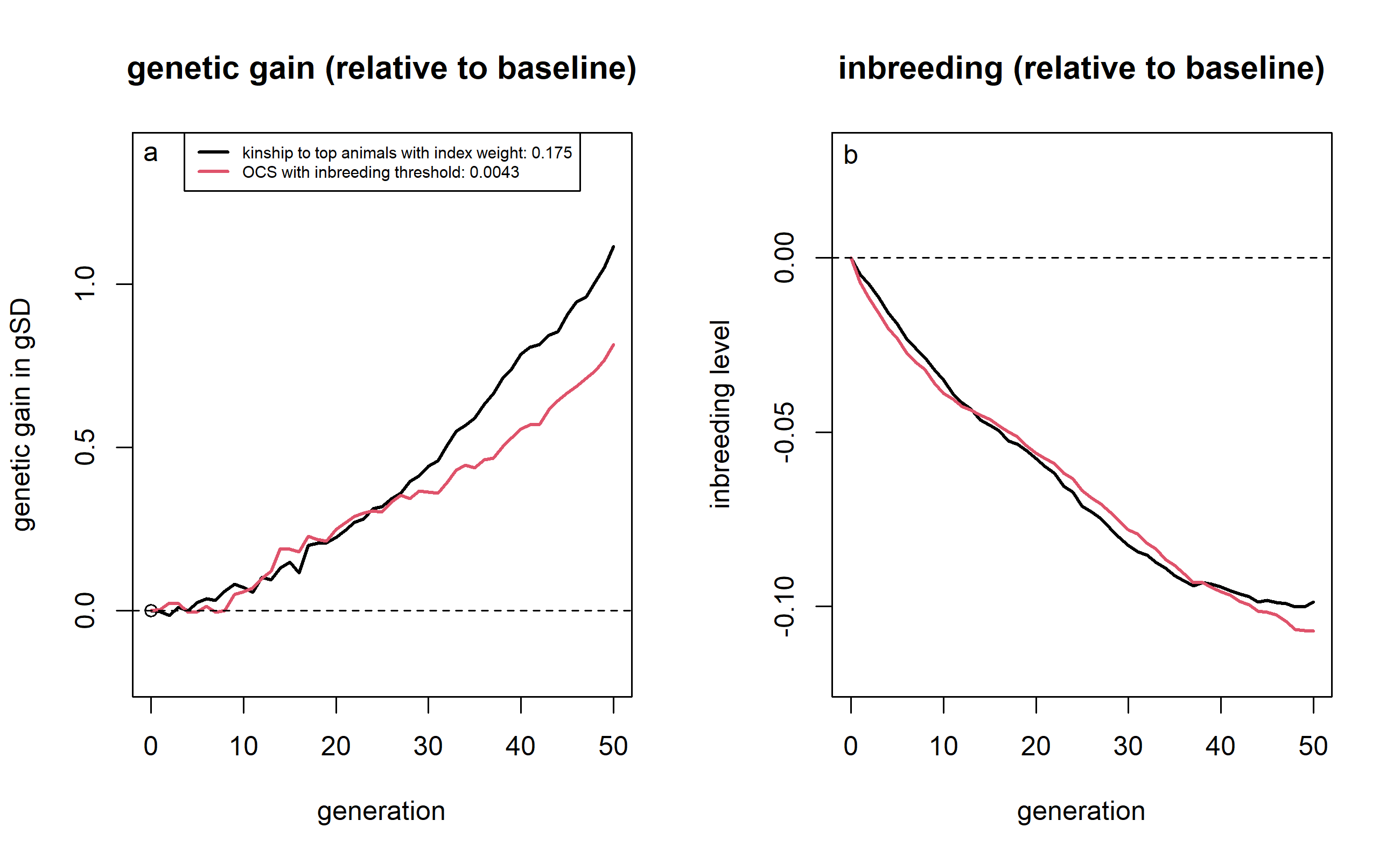

Supplement: Supplementary file 14 — Supplementary material 14 Comparison of genetic gains and inbreeding levels between optimum contribution selection and kinship in selection. Genetic gains (a) and inbreeding levels (b) relative to selection based on estimated breeding values when using optimum contribution selection with an maximum inbreeding of 0.43% compared to the use of average kinship to top individuals as a trait in the selection index with a weight of 17.5%. [file 12711_2026_1034_MOESM14_ESM.png]
